# Day of Arrival as a Determinant of Endoscopy Timing in Non-Variceal Upper Gastrointestinal Bleeding: A Natural-Experiment Cohort Study of Clinical Outcomes and Length of Stay

**DOI:** 10.3390/jcm15207820

**Published:** 2026-10-10

**Authors:** Lokman Bayram, Faruk Danış

**Affiliations:** 1 Department of Emergency Medicine, Faculty of Medicine, Bolu Abant İzzet Baysal University, 14030 Bolu, Türkiye; lokki.bayram@gmail.com; 2 Department of Emergency Medicine, Bolu İzzet Baysal Training and Research Hospital, 14030 Bolu, Türkiye

**Keywords:** upper gastrointestinal bleeding, endoscopy, time to treatment, hospital emergency service, instrumental-variable analysis, length of stay, propensity score, cohort studies

## Abstract

**Background/Objectives:** Guidelines recommend endoscopy within 24 h in acute upper gastrointestinal bleeding, but observational studies are confounded by indication. We used the weekday endoscopy schedule of our centre as a natural experiment. **Methods:** We studied a retrospective cohort of adults undergoing endoscopy for non-variceal upper gastrointestinal bleeding at one tertiary emergency department from 2019 to 2025. Timing was urgent (<12 h), early (12 to 24 h) or elective (>24 h); the primary outcome was 30-day mortality. Friday or Saturday arrival served as an instrument for delay, and crude, multivariable, propensity-based and instrumental-variable estimates were compared. **Results:** Among 413 episodes in 387 patients, median time to endoscopy was 22.1 h, with 54.5% within 24 h. The groups did not differ on 24 recorded baseline variables or six risk scores. Thirty-day mortality was 5.3% (22 deaths), with no detectable difference by timing (4.2%, 3.9%, and 6.9%; *p* = 0.420; adjusted odds ratio, elective versus early: 1.42, 95% CI: 0.50 to 4.03). Rebleeding, repeat endoscopy, intensive care admission and a composite outcome were unrelated. Total stay was longer with delay (incidence rate ratio: 1.40, 95% CI: 1.14 to 1.73), while stay from endoscopy onward was not (1.05, 95% CI: 0.80 to 1.38), and each day of waiting added 1.03 days of admission (95% CI: 0.60 to 1.46). Friday or Saturday arrival raised the elective proportion from 33.7% to 87.8% without altering the measured severity. **Conclusions:** Delay beyond 24 h was associated with no detectable difference in outcomes, although the 22 deaths left a moderate effect possible. The additional bed-days equalled the wait itself, which arose after gastroenterology consultation.

## 1. Introduction

Two major societies disagree on how confident clinicians should be about endoscopy timing in acute upper gastrointestinal bleeding. The European Society of Gastrointestinal Endoscopy recommends endoscopy within 24 h, and its 2026 update explicitly does not recommend endoscopy within 6 or 12 h unless haemodynamic instability persists after adequate resuscitation [1,2]. The American College of Gastroenterology suggests the same 24 h window but rates the evidence as low [3]. Among 516 high-risk patients, endoscopy within 6 h of consultation gave a 30-day mortality of 8.9% versus 6.6% for endoscopy at 6 to 24 h, with a confidence interval crossing zero [4]. A meta-analysis of five trials, 926 patients, found no benefit for any outcome [5].

Observational studies disagree instructively. A territory-wide cohort of 6474 patients found higher mortality after urgent endoscopy [6], a meta-analysis of 25 studies reached the same conclusion below 12 h [7], and among 120,835 geriatric patients, earlier endoscopy carried an odds ratio for death of 1.32 [8]. Yet a systematic review of more than 1.8 million patients returned a risk ratio of 0.97 [9], and in propensity-matched cohorts it was found that timing was not an independent predictor [10]. Confounding by indication is the likeliest explanation. The clinician deciding when to scope also sees how sick the patient is, so timing groups in most cohorts are not comparable. In 2525 prospectively studied patients, bleeding severity drove both the timing decision and the outcome [11].

Attempts to break that circularity have relied on propensity scores and inverse probability weighting [6,10,12], which balance only what has been measured. What the field lacks is variation in timing demonstrably unrelated to the patient’s condition. Randomisation creates this, and an instrumental variable can approximate it [13,14,15]. A second gap is arithmetical, not causal. Length of stay is repeatedly reported as an outcome of endoscopy timing [16,17], yet total stay contains the pre-endoscopy wait by construction, so a delayed group is guaranteed to record a longer admission whether or not its recovery is worse. We did not identify a study that separated the two components.

In many hospitals, including ours, endoscopy runs on weekdays, so arrival day largely determines waiting time. That creates a natural experiment. We used arrival on Friday or Saturday as an instrumental variable for delayed endoscopy in a cohort with non-variceal bleeding, tested whether timing was associated with mortality and five other outcomes across five analytic approaches, split the waiting interval into emergency department and post-consultation components, and separated total length of stay into the wait and the recovery after it.

## 2. Materials and Methods

### 2.1. Study Design and Setting

This retrospective cohort study was conducted in the emergency department of İzzet Baysal Training and Research Hospital, Bolu Abant İzzet Baysal University, a tertiary referral centre in northwestern Türkiye handling about 120,000 visits each year. Gastroenterology cover follows an on-call rota. Endoscopy takes place in a dedicated weekday unit, which also carries the outpatient programme. Reporting follows STROBE [18]. The data for this study were obtained from the same patient cohort as a residency (medical specialty) thesis completed at the Department of Emergency Medicine, Faculty of Medicine, Bolu Abant İzzet Baysal University.

### 2.2. Participants

We screened all adults evaluated in the emergency department for suspected upper gastrointestinal bleeding between April 2019 and December 2025, defined by haematemesis, coffee ground vomiting, melena or haematochezia with a compatible picture. Episodes entered the cohort only if oesophagogastroduodenoscopy occurred during the index admission. Variceal bleeding was excluded, since it follows a different pathway and guidelines.

Screening identified 800 assessments, of which 369 had no endoscopy during the index visit. Six of the remaining 431 records were duplicate entries of the same procedure and were removed. Twelve variceal episodes were then excluded. That left 413 episodes in 387 patients.

The 369 patients without endoscopy require comment, since the decision to scope is itself part of management. The exposure of this study is undefined when no procedure occurs, so these patients cannot enter a timing group. They were heterogeneous: some presentations did not support the diagnosis on evaluation, some patients were judged too low in risk for inpatient endoscopy, some declined or were transferred, and some died or were discharged before a procedure could take place. The ethics approval (protocol 2026/249) and the institutional data permission covered the endoscopy cohort only. Individual-level data on the 369 were not extracted, and obtaining them now would require a new ethics application and a new data release. Their absence could bias the comparison in a known direction, because a patient who deteriorated or died while waiting over a weekend would never enter the delayed group. We treat this as a limitation and quantify how many unobserved events would overturn each null result (Section 2.7).

Report text was retrievable for 412 of 413 episodes, so variceal-source, duplicate-record, therapy and haemostasis checks were adjudicated cohort-wide at report level rather than from coded fields. One episode lacked report text; its coded diagnosis, Forrest class and therapy fields were complete and kept as recorded.

Twenty-three patients presented with more than one bleeding episode, contributing 26 episodes beyond their first. All 413 episodes formed the primary analysis set, with clustering on patient handled as described below and the first episode per patient treated as a prespecified sensitivity analysis (*n* = 387).

### 2.3. Exposure

The exposure, time from arrival to endoscopy start, came from hospital information system timestamps, not from a derived field. Patients were grouped as urgent (less than 12 h), early (12 to 24 h) or elective (more than 24 h), with early as the reference in every model, since both European and American guidelines place their recommended window there [1,3]. We also recorded arrival to consultation request and request to endoscopy, so delay could be attributed to a phase of care.

Three alternative groupings were prespecified for sensitivity analysis: the four-tier scheme implied by the 2026 European guideline (6 h or less, more than 6 to 12 h, more than 12 to 24 h, more than 24 h); the scheme of Guo and colleagues (6 h or less, more than 6 to 24 h, more than 24 to 48 h, more than 48 h) [6]; and the consultation request in place of arrival as time zero.

### 2.4. Outcomes

The primary outcome was 30-day all-cause mortality, counted from arrival rather than endoscopy. Secondary outcomes included in-hospital mortality, rebleeding, repeat endoscopy, red-cell transfusion, intensive care unit admission and total length of stay. A prespecified composite outcome comprised 30-day mortality, rebleeding, repeat endoscopy, surgery or interventional radiology, whichever occurred first. Haemostasis success was assessed only among the 115 patients who received endoscopic therapy. Transfusion was recorded as any red-cell transfusion during admission, along with the number of units. Because the records did not time each unit relative to the procedure, transfusion before and after endoscopy could not be separated.

Total length of stay contains the pre-endoscopy wait by construction, so a group waiting longer records a longer stay even if nothing else differs. We derived post-endoscopy length of stay as total stay minus the interval to endoscopy, in days. In 25 episodes the recorded stay was shorter than the wait, so no post-endoscopy interval could be computed: 16 were urgent endoscopies with a recorded stay of zero days, discharged on the day of admission while the procedure sat hours into the visit, and 9 were elective. Every post-endoscopy result reported here, descriptive and modelled, and the total-stay comparison used in the decomposition, come from the same 388 episodes. Because a derived difference is mathematically coupled to its components, the decomposition is checked against analyses that use only measured quantities (Section 2.7).

### 2.5. Variables and Risk Scores

One investigator abstracted demographics, vital signs, symptoms, laboratory results, comorbidities and antithrombotic use from the hospital information system using a standardised form. Endoscopic diagnosis, Forrest class, therapy type and haemostasis outcome came from the endoscopy report; shock index and comorbidity count were derived. Before the analysis set was frozen, the dataset was audited cell by cell against the report text and nine single-cell coding inconsistencies were corrected.

Six pre-endoscopy risk scores were computed from raw clinical variables: Glasgow-Blatchford, AIMS65, ABC [19], MAP(ASH), the T-Score and the admission Rockall score, each recomputed from its components rather than read from a stored field. Two can be operationalised in more than one way, and both versions are compared. The recomputed ABC score matched the version using the published creatinine thresholds in 349 of 413 episodes, and AIMS65 was unchanged in 396 of 413 at a systolic blood pressure criterion of 90 mmHg or below rather than below 90 mmHg (Table S11). Scores enter only to test baseline severity and to supply the adjustment variable; their comparative discrimination [20,21] is outside the scope of this report.

### 2.6. The Natural Experiment and Instrumental Variable Analysis

Arrival on Friday or Saturday was used as an instrumental variable for delayed endoscopy, because endoscopy services here run almost entirely during weekday hours, so arrival day sets much of the waiting time. This wait is largely independent of the patient’s condition.

An instrument must shift the exposure, be independent of the outcome except through the exposure, and be unrelated to unmeasured confounders [13,14]. The first was tested with the first-stage F statistic. The second could not be proven but was probed by comparing baseline severity across levels of the instrument. We report the reduced-form association with each outcome, a two-stage least squares estimate, and the limits of what the instrument resolves.

### 2.7. Statistical Analysis

The analysis plan was locked before any effect estimate existed. It separated a confirmatory layer from an exploratory one. The confirmatory layer comprised the primary outcome and six secondary outcomes, all named in advance in the ethics application. Subgroup analyses, interaction tests, instrumental-variable estimates, the spline, the post-endoscopy decomposition, and the analyses added at peer review were all treated as exploratory, and each one is labelled as such throughout the manuscript.

Continuous variables are reported as median with interquartile range and compared using the Kruskal–Wallis test. Categorical variables were counts and percentages, compared with chi-square or Fisher’s exact test when an expected cell count fell below five. Pairwise contrasts between the three timing groups are reported for every outcome as Fisher exact odds ratios, Wald risk differences and Hodges–Lehmann shifts, with Holm adjustment within each outcome. Multivariable logistic regression estimated adjusted odds ratios against the early group. Mortality, intensive care admission and the composite were adjusted for the ABC score; age was not entered separately, being a component of that score, and a sensitivity analysis included both. Rebleeding and repeat endoscopy were additionally adjusted for a high-risk Forrest class and endoscopic therapy; rebleeding was also adjusted for antithrombotic use and transfusion for admission haemoglobin, antithrombotic use and sex. Because 23 patients contributed more than one episode, every logistic model was re-estimated by generalised estimating equations with an independence working correlation and patient as the cluster, and the stay models used cluster-robust standard errors; the clustered estimates are those reported. Firth penalised regression confirmed both mortality models. Surgery and interventional radiology were not modelled separately. Length of stay, total and post-endoscopy, was modelled with negative binomial regression. Predicted 30-day mortality for each timing interval was obtained from the ABC-adjusted model by marginal standardisation, with bootstrap percentile intervals from 2000 resamples.

For the causal layer we estimated a propensity score for the timing group from age, sex, ABC score, Glasgow-Blatchford score, ASA class of III or higher, Charlson index, systolic blood pressure, admission haemoglobin, haematemesis and antithrombotic use. Inverse probability of treatment weighting was applied, with balance judged by standardised differences below 0.10 [12]. Matching was one-to-one nearest neighbour with calliper 0.2 standard deviations of the logit, and performed separately for urgent versus early and for elective versus early, with standard errors clustered on matched pairs. Time to endoscopy was also modelled continuously with a restricted cubic spline at three knots.

Because a derived post-endoscopy interval was coupled to the wait by arithmetic, the decomposition was checked with analyses that used only the measured quantities. The slope of measured total stay on measured waiting time, in days per day, was estimated by ordinary least squares with cluster-robust standard errors; by median regression, in waits of 5 days or less; and by two-stage least squares with the arrival-day instrument. A slope of 1 means each day of waiting adds one day of admission and nothing else; a slope above 1 would mean that waiting also prolongs recovery. The post-endoscopy result was also re-estimated with all 413 episodes retained and negative intervals set to zero, and as a discharge-hazard model.

Selection at the endoscopy decision was examined by tipping-point analysis. This method estimated the number of deaths that would need to be added to the delayed group, or to Friday or Saturday arrivals, for each null comparison to become significant. A scenario table then combined the 413 scoped episodes with the 369 unscoped patients, applying assumed mortality and weekend excess to the latter group. A sensitivity analysis excluded arrivals in 2020 and 2021, the years most affected by the COVID-19 pandemic.

The primary outcome had no multiplicity adjustment. The seven confirmatory secondary outcomes were adjusted using the Benjamini–Hochberg procedure with unadjusted values alongside; exploratory analyses remained unadjusted. Missing data were limited to lactate in 6 episodes (1.5%), which entered no model, so no imputation was performed and all analyses were complete-case.

Analyses were performed in IBM SPSS Statistics for Macintosh, version 29.0 (IBM Corp., Armonk, NY, USA), and in R version 4.6.1 (R Foundation for Statistical Computing, Vienna, Austria) within RStudio version 2026.09.0+174 (Posit Software, PBC, Boston, MA, USA). Descriptive statistics and between-group comparisons were computed in SPSS; propensity-score weighting and matching (MatchIt 4.7.2, WeightIt 2.0.0, cobalt 5.0.0), generalised estimating equations (geepack 1.3.13), penalised regression (logistf 1.26.1), negative binomial regression (MASS 7.3-66), splines (rms 8.1-1), survival models (survival 3.8-11) and the instrumental-variable estimates (AER 1.2-17, sandwich 3.1-3) were computed in R. Every reported quantity was recomputed by a second, independently written script before entering the manuscript. Two-sided *p* values below 0.05 were considered significant.

## 3. Results

### 3.1. Cohort

**Figure 1:**
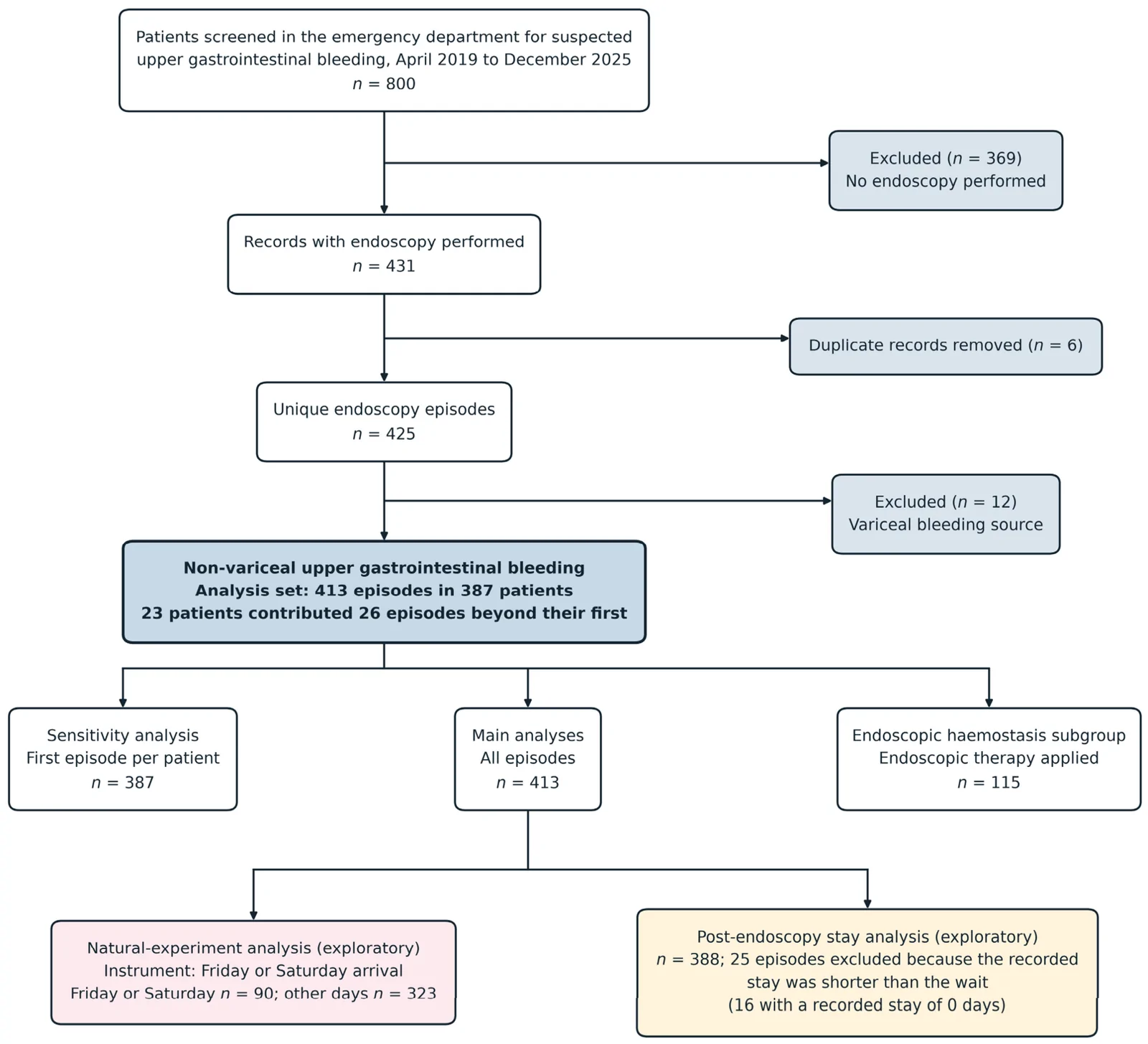
Participant flow through the study, following the STROBE recommendation for reporting the selection of participants. Of 800 patients assessed in the emergency department of İzzet Baysal Training and Research Hospital, Bolu Abant İzzet Baysal University, Türkiye, for suspected upper gastrointestinal bleeding between April 2019 and December 2025, 369 were excluded because no endoscopy was performed, leaving 431 records; 6 duplicate records were removed, giving 425 unique endoscopy episodes, of which 12 were excluded because the bleeding source was variceal. The final analysis set comprises 413 episodes contributed by 387 patients, 23 of whom contributed 26 further episodes beyond their first. Main analyses use all 413 episodes; a sensitivity analysis restricted to the first episode per patient uses 387 episodes; the endoscopic haemostasis subgroup comprises the 115 episodes in which endoscopic therapy was applied. Two exploratory analyses branch from the main analysis set: the natural-experiment analysis, which uses Friday or Saturday arrival as the instrument (90 episodes on Friday or Saturday, 323 on other days), and the post-endoscopy stay analysis, which uses 388 episodes after excluding 25 in which the recorded stay was shorter than the recorded wait, 16 of which had a recorded stay of 0 days. STROBE, Strengthening the Reporting of Observational Studies in Epidemiology; *n*, number of episodes unless patients are named.

The analysis set comprised 413 episodes in 387 patients (Figure 1). The most common endoscopic diagnosis was peptic ulcer in 167 episodes (40.4%), then erosive gastritis or duodenitis in 130 (31.5%); no source was identified in 38 (9.2%) and malignancy accounted for 27 (6.5%). A high-risk Forrest lesion (Ia, Ib, IIa or IIb) was present in 95 (23.0%) and endoscopic therapy was applied in 115 (27.8%), most often clips alone in 61 (14.8%) (Tables S8–S10). Table 1 gives the baseline characteristics.

### 3.2. Timing and Where the Delay Arises

**Figure 2:**
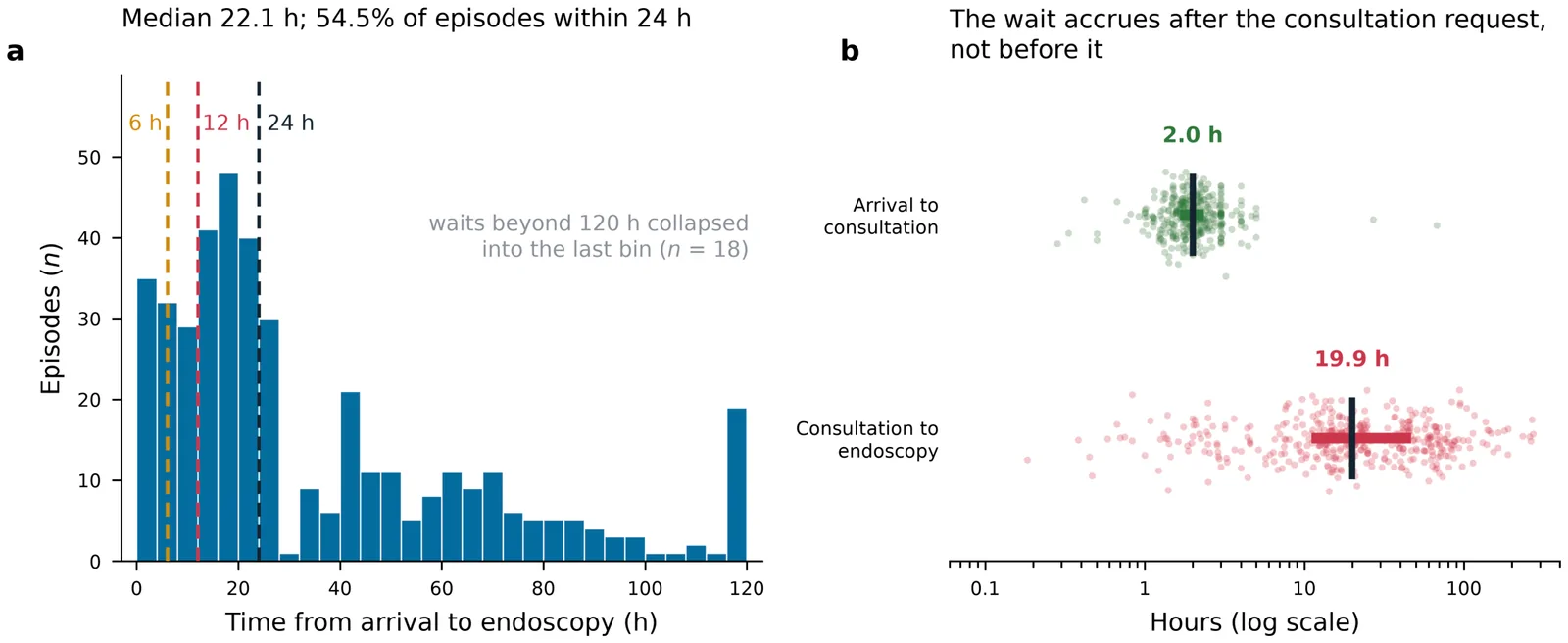
Time from emergency department arrival to endoscopy and its decomposition into the two process intervals in all 413 episodes. Panel (**a**): Distribution of the interval from arrival to endoscopy in 4 h bins, with dashed reference lines at the 6 h, 12 h and 24 h thresholds used in the current guidance; the 18 episodes with waits longer than 120 h are collapsed into the final bin so that the body of the distribution remains readable. The median wait was 22.1 h and 54.5% of episodes reached endoscopy within 24 h. Panel (**b**): The same waiting time split at the gastroenterology consultation request on a logarithmic hour scale; each dot is one episode, the horizontal bar is the interquartile range and the vertical rule is the median. The interval from arrival to the consultation request was short and tightly distributed (median 2.0 h, interquartile range 1.7 to 2.3 h), whereas the interval from consultation request to endoscopy was long and widely dispersed (median 19.9 h, interquartile range 11.1 to 46.7 h), so the wait accrued after the emergency department completed its part of the pathway. h, hours; *n*, number of episodes.

Median time from arrival to endoscopy was 22.1 h (IQR 13.2 to 49.5). Just over half of the episodes, 225 of 413 (54.5%), were managed within 24 h, and 406 (98.3%) were done in weekday working hours. The delay sits outside the emergency department. From arrival to the gastroenterology consultation request took a median of 2.0 h (IQR 1.7 to 2.3) and from that request to endoscopy 19.9 h (IQR 11.1 to 46.7), with the two intervals differing (Wilcoxon signed-rank *p* < 0.001). Table 2 gives both, and Figure 2 shows their distributions.

### 3.3. Baseline Comparability of the Timing Groups

Endoscopy was performed urgently in 96 episodes, early in 129 and electively in 188. None of the 24 baseline variables in Table 1 differed across groups on the measured data, and the smallest *p* value, 0.056, was recorded for the Glasgow Coma Scale. Its median was 15 in every group. Six risk scores and the Charlson index were also similar across groups, with *p* values ranging from 0.211 to 0.845 (Table S1).

Comparability on recorded variables is not the same as comparability in severity. Endoscopic therapy was applied in 38 of 96 urgent episodes (39.6%), 36 of 129 early episodes (27.9%) and 41 of 188 elective episodes (21.8%) (*p* = 0.007). High-risk Forrest lesions showed the same trend, although they did not reach significance (31.2%, 21.7%, 19.7%; *p* = 0.083). Part of this gradient is expected from the natural history of ulcer bleeding: lesions that stop spontaneously tend to present a lower Forrest class when examined later. Another part may reflect a dimension of severity that the recorded variables and scores did not capture, one that drew clinicians towards earlier endoscopy. The groups should therefore be read as balanced on measured covariates, not as clinically equivalent. Haemostasis succeeded in 108 of 115 treated episodes (93.9%). Three of 95 high-risk lesions received no endoscopic therapy.

### 3.4. Clinical Outcomes

Thirty-day mortality occurred in 22 of 413 (5.3%) and did not differ detectably by timing group: 4.2%, 3.9% and 6.9% (*p* = 0.420). Rebleeding occurred in 49 episodes (11.9%) at near-identical rates across groups (*p* = 0.976). Repeat endoscopy and intensive care admission showed no gradient either. The composite outcome followed the same pattern (Table 3). Surgery and interventional radiology were each required once.

Two outcomes varied. Red-cell transfusion occurred in 193 episodes (46.7%): 34.4%, 48.8% and 51.6% across groups (*p* = 0.019, adjusted *p* = 0.096). Total length of stay differed more robustly, with medians of 3, 4 and 5 days (*p* < 0.001, adjusted *p* < 0.001).

Table 4 reports every pairwise contrast between the three timeframes. For 30-day mortality, the elective-versus-early risk difference was 3.0 percentage points (95% CI −1.9 to 8.0; odds ratio 1.84, 95% CI 0.60 to 6.76; Holm-adjusted *p* = 0.979). This interval includes both no effect and a near doubling. Transfusion was the only binary outcome with a pairwise contrast below 0.05 before adjustment. These were urgent versus early and urgent versus elective, and neither survived Holm adjustment. The two stay outcomes behaved differently from each other. For total stay, the elective group separated from both other groups. For post-endoscopy stay, it separated from neither.

### 3.5. Adjusted and Causal Estimates

**Figure 3:**
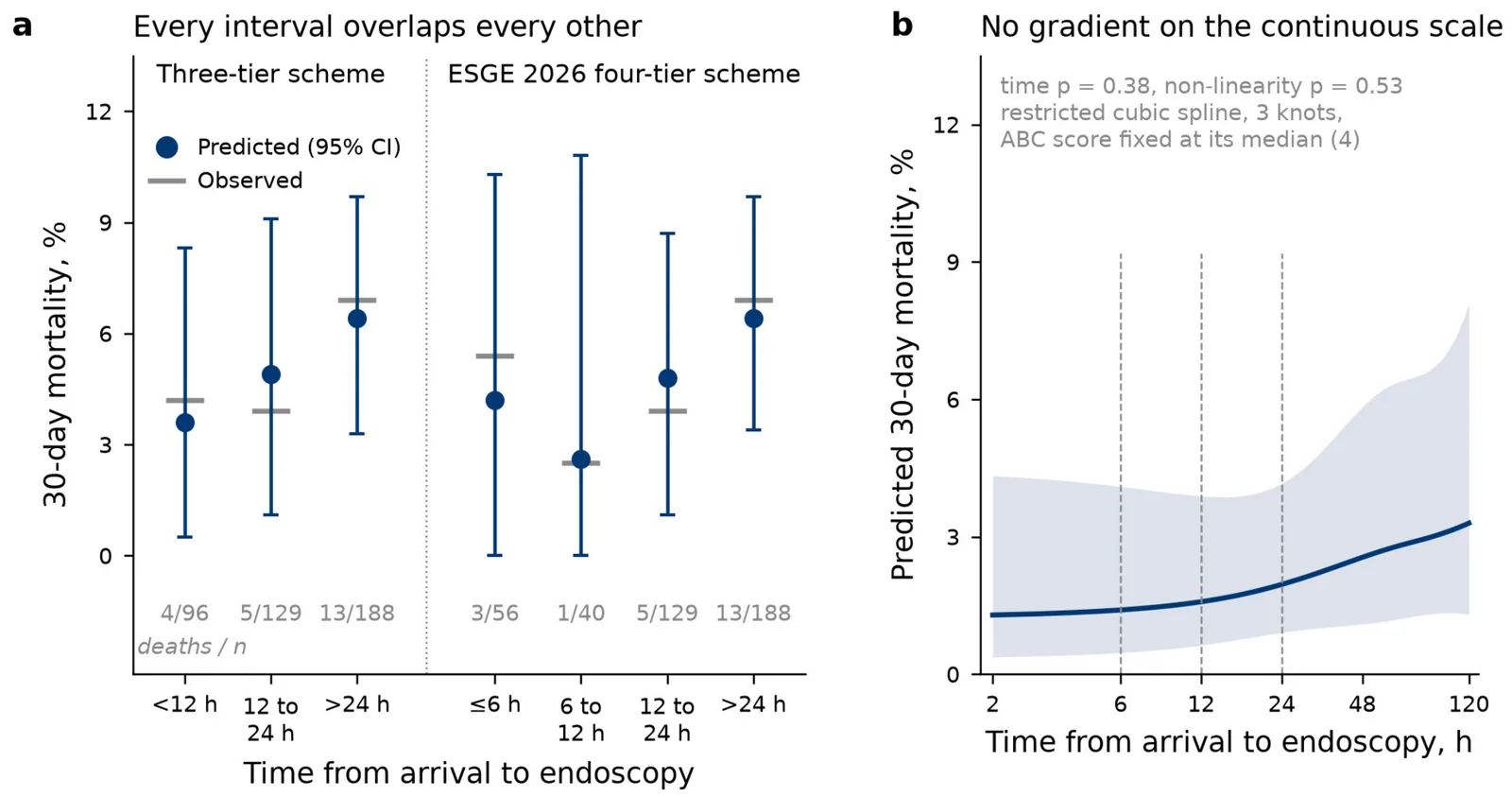
Predicted and observed 30-day mortality by endoscopy timeframe, requested at peer review. Panel (**a**): Observed 30-day mortality (grey bar) and the mortality predicted from the ABC-adjusted logistic model by marginal standardisation to the whole cohort (navy point, bootstrap percentile 95% CI from 2000 resamples) for the three-tier scheme used in this study and for the four-tier scheme implied by the 2026 European guideline; deaths and group sizes are printed under each interval. Every confidence interval overlaps every other under both schemes. Panel (**b**): Predicted 30-day mortality across the continuous time from arrival to endoscopy from a logistic model with a restricted cubic spline at three knots and ABC score fixed at its median of 4, with the 95% confidence band shaded and the 6 h, 12 h and 24 h thresholds marked; the overall test for time gives *p* = 0.38 and the test for non-linearity *p* = 0.53. The time axis is logarithmic. ABC, age, blood tests and comorbidities score; CI, confidence interval; ESGE, European Society of Gastrointestinal Endoscopy; h, hours; *n*, number of episodes.

After adjustment for the ABC score, with standard errors clustered on patients, neither urgent nor elective timing was associated with 30-day mortality (odds ratios of 0.69, 95% CI 0.13 to 3.71, and 1.42, 95% CI 0.50 to 4.03). Firth penalised regression agreed (0.73 and 1.36; Table S7). Accounting for the 23 patients with repeat episodes changed no point estimate; standard errors altered by 1% to 14% (Table S13). The ABC score itself predicted mortality strongly at 1.48 per point (95% CI 1.28 to 1.71). Rebleeding, repeat endoscopy, intensive care admission and the composite outcome were unrelated to timing in the widened models (Table 5). Figure 3 shows the ABC-adjusted predicted 30-day mortality for each timeframe: 3.6% (95% CI 0.5 to 8.3) for urgent, 4.9% (1.1 to 9.1) for early and 6.4% (3.3 to 9.7) for elective endoscopy. Every interval overlapped every other under both the three-tier and four-tier scheme. No gradient appeared on the continuous scale (*p* = 0.38, non-linearity *p* = 0.53).

**Figure 4:**
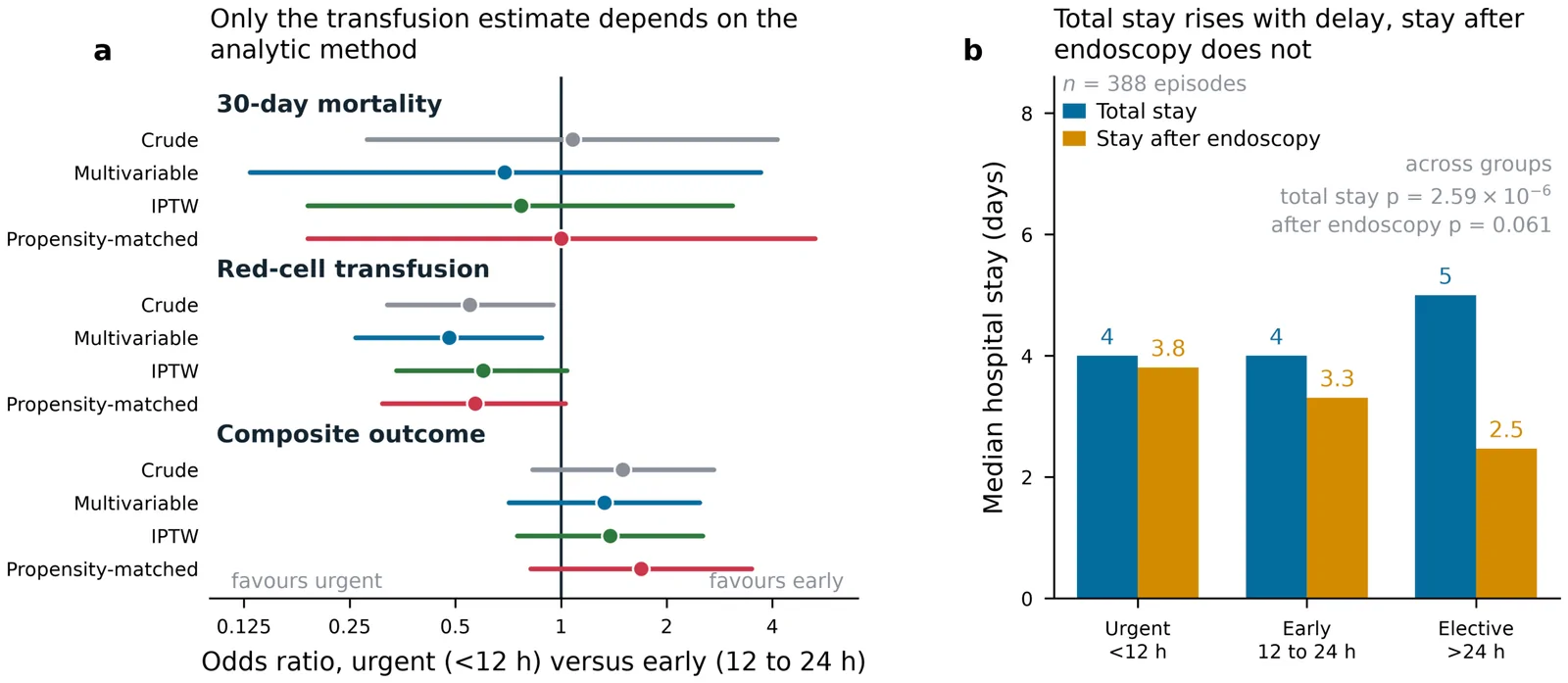
Effect estimates for endoscopy timing. Panel (**a**): Odds ratios with 95% confidence intervals for 30-day mortality, red-cell transfusion and the composite outcome, comparing urgent endoscopy (<12 h) with early endoscopy (12 to 24 h) under four analytic approaches: crude (unadjusted), multivariable logistic regression (adjusted for the ABC score, and for transfusion, additionally adjusted for admission haemoglobin, antithrombotic use and sex), inverse probability of treatment weighting and propensity-score matching (176 matched episodes). The axis is a logarithmic odds ratio scale and the vertical rule marks an odds ratio of 1, that is, no difference; points to the left of the rule favour urgent endoscopy. Crude odds ratios are unadjusted estimates with Wald 95% confidence intervals; the other three sets of estimates come from the corresponding multivariable, weighted and matched analyses. Every interval for 30-day mortality and for the composite outcome includes 1 under all four approaches, whereas for red-cell transfusion the crude and multivariable intervals exclude 1 and the weighted and matched intervals include it; the transfusion association is therefore method-dependent and does not survive correction for multiple comparisons. Panel (**b**): Median total hospital stay and median stay measured from endoscopy onward, both reported on the same 388 episodes for which a stay after endoscopy can be computed (80 urgent, 129 early, 179 elective); *p* values are for the comparison across the three timing groups. Twenty-five episodes are excluded from both bars because the recorded stay was shorter than the recorded wait, 16 of them urgent endoscopies whose recorded stay was 0 days and 9 elective; reporting the two intervals on a common denominator avoids the artefact that arises when total stay is taken from all 413 episodes and the stay after endoscopy from the 388. On this common denominator, total stay rises across the timing groups (medians 4, 4 and 5 days) while the stay measured from endoscopy onward does not (medians 3.81, 3.31 and 2.47 days), indicating that the additional bed-days are the waiting time itself rather than a slower recovery. OR, odds ratio; CI, confidence interval; IPTW, inverse probability of treatment weighting; h, hours; *n*, number of episodes.

The transfusion association varied by method; Figure 4 shows the estimates together. Urgent endoscopy was associated with less transfusion in the crude analysis (odds ratio 0.55, 95% CI 0.32 to 0.95) and after adjustment for the ABC score, admission haemoglobin, antithrombotic use and sex (0.48, 95% CI 0.26 to 0.88). This association then weakened. Inverse probability weighting gave 0.60 (95% CI 0.34 to 1.04), and matching of 176 episodes gave 0.57 (95% CI 0.31 to 1.03); neither excluded 1. The instrumental-variable estimate was a risk difference of 0.025 (95% CI −0.192 to 0.241). Weighting balanced covariates well (maximum standardised difference of 0.036 continuous and 0.017 binary; Tables S6 and S12). The attenuation is therefore not a balance artefact. Transfusion also stepped up at 12 h rather than rising steadily, recorded at 35.7%, 32.5%, 48.8% and 51.6% across the four tiers.

The elective-versus-early comparison was also estimated by matching and weighting (Tables S14 and S15). In 124 matched pairs, balanced to a largest standardised difference of 0.091, transfusion, rebleeding, intensive care admission and the composite did not differ, total stay was longer (incidence rate ratio 1.43, 95% CI 1.13 to 1.81) and post-endoscopy stay was not (1.09, 95% CI 0.79 to 1.49). For 30-day mortality, the matched estimate was 2.92 (95% CI 1.04 to 8.17), resting on 11 deaths against 4 (9 discordant pairs against 2; exact McNemar *p* = 0.065; Firth estimate 3.09, 95% CI 0.91 to 12.64), whereas weighting on the same covariates gave 1.17 (95% CI 0.39 to 3.54). We report both. Neither is precise. An estimate that moves from 1.2 to 2.9 with the choice of method, on 15 deaths, describes precision rather than delay, and it is the clearest reason in these data to read the mortality result as undetected rather than absent.

Length of stay moved consistently in two parts. On the 388 episodes with a computable post-endoscopy interval (80 urgent, 129 early, 179 elective), median total stay was 4.00 days (IQR 2.00 to 6.25), 4.00 days (IQR 2.00 to 6.00) and 5.00 days (IQR 4.00 to 8.50) across the three timing groups (*p* < 0.001). Median stay measured from endoscopy onward was 3.81 days (IQR 1.88 to 6.03), 3.31 days (IQR 1.49 to 5.35) and 2.47 days (IQR 1.25 to 5.25) (*p* = 0.061, exploratory). Elective timing carried an incidence rate ratio of 1.40 for total stay (95% CI 1.14 to 1.73, *p* = 0.001) and 1.05 for post-endoscopy stay (95% CI 0.80 to 1.38, *p* = 0.707), with patient-clustered standard errors.

The post-endoscopy interval is coupled to the wait by construction: had total stay been unrelated to waiting, the derived interval would have correlated with the wait at r = −0.23 through arithmetic alone. The observed correlation was 0.01, and the covariance of wait and total stay (3.24 days squared) almost equalled the variance of the wait (3.13 days squared), which is what a one-to-one carry-over produces. We tested that carry-over on the two measured quantities. Each day of waiting added 1.03 days of admission (95% CI 0.60 to 1.46; *p* = 0.90 against a slope of exactly 1); the unadjusted, median-regression, short-wait and instrumented slopes were 1.09, 0.88, 0.75 and 0.48 days, and none exceeded 1 (Table S16). With the continuous wait in the model, the elective indicator fell to 1.09 (95% CI 0.89 to 1.34). Retaining all 413 episodes with negative intervals set to zero gave an elective rate ratio of 1.00 (95% CI 0.79 to 1.26), and the hazard of discharge after endoscopy did not differ (hazard ratio 1.03, 95% CI 0.82 to 1.29). Of the 25 excluded episodes, 16 were urgent and 9 elective, with a lower median Glasgow-Blatchford score (8 versus 11) and no deaths; their exclusion removed short stays from the urgent group and worked against the post-endoscopy finding rather than for it.

### 3.6. Findings of the Natural Experiment

**Figure 5:**
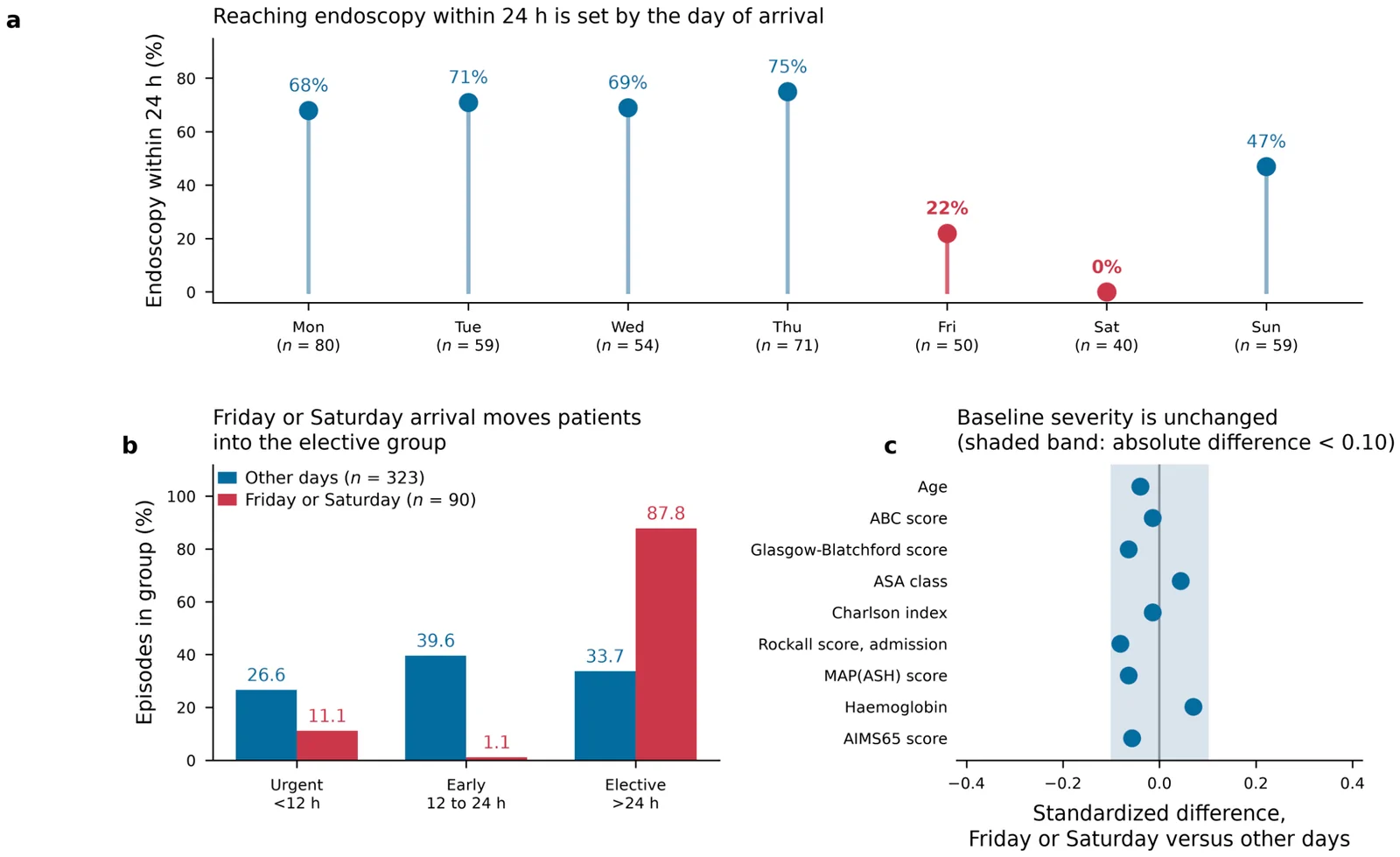
The natural experiment created by the weekday endoscopy schedule. Panel (**a**): Percentage of episodes reaching endoscopy within 24 h according to the day of arrival, with Friday and Saturday marked in red; the number of episodes arriving on each day is given under the day label. The lowest value on any of Monday to Thursday (68%) exceeds the highest value on Friday, Saturday or Sunday (47%), and no patient arriving on a Saturday reached endoscopy within 24 h, which is drawn as a marker at the zero line. Panel (**b**): Distribution of episodes across the three timing groups for arrivals on Friday or Saturday (90 episodes) compared with arrivals on other days (323 episodes); Friday or Saturday arrival raises the elective share from 33.7% to 87.8% while reducing the urgent and early shares. Panel (**c**): Standardised differences in baseline severity between Friday or Saturday arrivals and arrivals on other days for all nine variables on which the exclusion restriction was tested; the shaded band marks an absolute standardised difference below 0.10, the conventional threshold for negligible imbalance. All nine standardised differences lie inside the band, the largest being 0.081 for the admission Rockall score, so the instrument shifts the timing of endoscopy without shifting the severity of the patients it acts on. ABC, age, blood tests and comorbidities score; AIMS65, score combining albumin, international normalised ratio, altered mental status, systolic blood pressure and age above 65 years; ASA, American Society of Anesthesiologists physical status class; Charlson index, Charlson comorbidity index; Glasgow-Blatchford score, Glasgow-Blatchford bleeding score; MAP(ASH) score, mortality, admission and prediction score based on albumin, international normalised ratio, mental status, systolic blood pressure, haemoglobin and age; Rockall score, admission, pre-endoscopy Rockall score; h, hours; *n*, number of episodes.

Timing tracked the day of arrival. Endoscopy within 24 h ranged from 68% to 75% Monday through Thursday, fell to 22% on Friday, and was zero for all 40 Saturday arrivals, recovering to 47% on Sunday (Figure 5, Table S5). Friday or Saturday arrival raised the elective proportion from 33.7% to 87.8% and lowered the urgent proportion from 26.6% to 11.1%. First-stage F statistics were 39.02 for hours to endoscopy and 103.16 for elective status.

None of the nine severity indicators differed across the instrument (*p* from 0.364 to 0.881, Table 6). In the reduced form, arrival on Friday or Saturday was unrelated to 30-day mortality (3.3% versus 5.9%, *p* = 0.435), rebleeding (13.3% versus 11.5%, *p* = 0.762), transfusion (47.8% versus 46.4%, *p* = 0.916) and the composite outcome (26.7% versus 25.1%, *p* = 0.866). Two-stage least squares estimates were null but wide: a risk difference of −0.047 for 30-day mortality (95% CI −0.132 to 0.037) and 0.025 for transfusion (95% CI −0.192 to 0.241). For stay the instrumented estimates were 1.05 days for total stay (95% CI −3.12 to 5.22) and −1.79 days for post-endoscopy stay (95% CI −6.10 to 2.53). Neither is informative; the length-of-stay conclusion rests on the group comparison and the slope analysis. Post-endoscopy stay was shorter after a Friday or Saturday arrival in the reduced form (median 2.25 versus 3.32 days, *p* = 0.003), but coupling pushed this derived interval in exactly that direction, so we note only that it was not longer.

### 3.7. Sensitivity Analyses

Conclusions held across all prespecified variations (Table S3): first episodes only (*n* = 387), daytime arrivals, the 2026 European four-tier scheme (Table S4), the Guo grouping, and consultation request as time zero. Transfusion *p* values ranged from 0.019 to 0.172, and mortality ranged from 0.407 to 0.640. The length-of-stay difference persisted in every variant, with *p* < 0.001 throughout. Post-endoscopy stay was non-significant in five of the six variants, with *p* from 0.054 to 0.145, but it reached *p* = 0.046 among daytime arrivals only. Modelling time continuously showed a transfusion association (*p* = 0.021), with no departure from linearity (*p* = 0.195). The mortality model also stayed linear, with *p* = 0.376.

Excluding the 69 episodes from 2020 and 2021 left 344 episodes with 21 deaths. Mortality did not differ by timing (*p* = 0.596; adjusted odds ratio for elective versus early 1.47, 95% CI 0.46 to 4.65). The total-stay difference persisted (*p* < 0.001; incidence rate ratio 1.31, 95% CI 1.09 to 1.58). The transfusion difference was borderline (*p* = 0.050), and the post-endoscopy stay difference reached *p* = 0.045, with the same ordering as in the full cohort. The instrument remained strong (first-stage F 67.99; elective 85.5% versus 34.9%) and was unrelated to mortality (2.9% versus 6.9%, *p* = 0.271).

Two episodes shared an arrival timestamp and identical admission laboratory values but appeared under different identifiers. The report text suggested that the second episode was a second-look endoscopy performed during the same admission, rather than a separate presentation. This could not be resolved. Both episodes were therefore retained, and the analysis was repeated after removing the later one. Nothing moved: 30-day mortality *p* changed from 0.420 to 0.413, transfusion *p* from 0.019 to 0.017, and total length of stay *p* from 6.0 × 10^−8^ to 6.8 × 10^−8^. Post-endoscopy stay *p* moved from 0.061 to 0.066, and the first-stage F for elective status moved from 103.2 to 101.5.

Among exploratory subgroups (Table S2a,b), only the ABC stratum modified the association between timing and 30-day mortality (interaction *p* = 0.048). The age interaction a Turkish cohort reported was not reproduced here (*p* = 0.421) [22].

### 3.8. How Much Selection Would Overturn the Null Results

The 369 patients who were not scoped are absent from every comparison. Eight additional deaths in the elective group, 2.2% of those 369 patients, would make the crude three-group mortality comparison significant and lift the lower limit of the elective-versus-early interval above 1. Nine additional deaths among Friday or Saturday arrivals would do the same for the reduced form, which would mean a 30-day mortality above 11% in the roughly 80 unscoped patients expected to have arrived on those days against 3.3% among those scoped (Table S17). Scenarios assigned the unscoped a 30-day mortality of 2% to 15% and a weekend excess of up to threefold. The combined reduced-form comparison stayed non-significant unless weekend arrivals who were not scoped died at least twice as often as weekday arrivals who were not scoped and mortality among the unscoped exceeded 10%. The null results are robust to moderate selection. They are fragile to strong selection. That is why they are reported as no detectable difference.

## 4. Discussion

In this cohort, the day of arrival largely set the waiting time before endoscopy. That wait was followed by a longer hospital admission but by no detectable difference in mortality, rebleeding, repeat endoscopy, intensive care admission, or a composite of adverse events. Two features of the study design shape how these results should be read. A weekday schedule, rather than a clinician’s judgement, allocated most of the waiting time, so the timing groups were balanced on 24 recorded variables and six risk scores. Total stay was also separated into the initial wait and the stay following the procedure. Both features carry limits.

The mortality result is a null that cannot be read as safety. With 22 deaths, the adjusted odds ratio for delay beyond 24 h was 1.42 (95% CI 0.50 to 4.03), the matched elective-versus-early estimate reached 2.92 on 15 deaths while the weighted estimate on the same covariates was 1.17, and the instrument, though strong, added no precision. The finding sits inside the randomised evidence. In 516 high-risk patients, 30-day mortality was 8.9% versus 6.6% at under 6 h versus 6 to 24 h [4]; five trials in 926 patients showed no benefit [5]; and a review of over 1.8 million patients returned a risk ratio of 0.97 (95% CI 0.74 to 1.27) [9]. Smaller cohorts found no association at 6 or 12 h thresholds [23,24,25], and where a signal appeared it pointed towards harm from urgent endoscopy [6,7,8,26,27,28], attributed to confounding by indication [11,15]. Our data are consistent with all of these and decisive for none; a moderate effect of delay on mortality in either direction remains possible.

The length-of-stay result is the finding this study adds. Siau and colleagues audited 348 patients across 20 centres at a median time to endoscopy of 21.2 h, close to our 22.1 h, and found that early endoscopy cut stay by a median of one day without affecting mortality [16]. Others report the same [17], and it is usually read as accelerated recovery. A simpler account fits our data, however. Each day of waiting added 1.03 days of admission (95% CI 0.60 to 1.46), a slope indistinguishable from 1 and estimated from two measured quantities without any derived variable. Conditioning total stay on the wait removed the elective effect. The hazard of discharge after endoscopy did not differ either. A derived post-endoscopy interval was mathematically coupled to the wait, so we quantified the direction and size of that coupling rather than relying on the derived interval alone. Had delay slowed recovery, the slope would have exceeded 1, and discharge after elective endoscopy would have been slower; neither happened. The bed-days attributable to delay appear to be the waiting itself. This is a single-centre observation. It needs confirmation.

Transfusion occurred more often after early and elective endoscopy than after urgent endoscopy. This association held with regression adjustment but not with weighting, matching, the instrument, or multiplicity correction. Two Turkish cohorts report differences in the same direction [17,24], and propensity matching in a third cohort removed timing as an independent predictor [10]. Because the records do not time each transfused unit relative to the procedure, transfusion given before endoscopy, which reflects the presentation, could not be separated from transfusion given after it. The association is therefore reported as method-dependent and not promoted.

The bottleneck was the gap between the decision to scope and an available list, not triage or resuscitation [29]. That gap is a property of the unit. In a prospective analysis of 1467 procedures, the number of scheduled outpatient cases independently predicted delay of inpatient endoscopy (odds ratio 6.76, 95% CI 2.24 to 20.35) [30]. Elsewhere, delay of inpatient endoscopy added a median of 2 bed-days, and unit or staff unavailability caused a quarter of the delays [31]. In a UK audit, the patients most often delayed arrived in the daytime through the emergency department when the lists were full [16], and 43% of English hospitals reported that routine workload impaired next-day endoscopy [32]. Weekend admission carried excess mortality only in centres without a weekend endoscopist [33], and a bleeding unit with protected capacity reported lower risk-standardised mortality than the national audit [34]. Programmed surveillance adds to the same lists: treat-to-target management of ulcerative colitis now schedules repeated endoscopic and histological assessment of mucosal healing [35,36]. The remedy, whether a protected inpatient slot or a seven-day service, had a cost beyond money, since out-of-hours haemostasis carried an adjusted odds ratio of 4.63 for adverse outcome in 301 patients [37].

Two caveats concern what the cohort contains. Endoscopic therapy was needed in 39.6% of urgent and 21.8% of elective episodes. Part of that gradient is the natural history of a lesion that stops while the patient waits, but part may be the severity the recorded variables did not capture, so the groups are balanced on measured covariates rather than clinically equivalent. And the cohort is one of peptic and mucosal disease. Rare lesions that bleed massively are not covered by timing guidance derived from ulcer bleeding [38], and they are too few here to inform it. Examples are Dieulafoy lesions, with 30-day rebleeding of 27% after matching against ulcers [39]; aortoenteric fistulae; haemobilia; and ampullary tumours, including a recently described ganglioneuroma coexisting with a pancreatic neuroendocrine tumour in which endoscopic haemostasis held for 36 h before surgery was required [40].

Arrival day is not a novel exposure, but to our knowledge it has not been used as an instrument, and as an instrument it isolates variation that the patient’s condition cannot have caused. External evidence supports the exclusion restriction. A multisite study of 7640 patients found no weekend effect on in-hospital mortality (adjusted odds ratio 1.06, 95% CI 0.83 to 1.35) [41], and an Italian registry of 2599 patients reported 5.6% versus 4.9% [42]. The one dissenting study attributed excess weekend rebleeding to endoscopist experience [43], which did not reproduce here. The results fit current guidance, which does not recommend endoscopy within 6 or 12 h unless haemodynamic instability persists after resuscitation [1,2,3], and support the 24 h target on resource grounds independent of mortality.

Limitations remain. The 369 patients who were not scoped are absent, and a patient who died or was discharged while waiting over a weekend could never enter the delayed group. Eight unobserved deaths in that group would overturn the crude mortality comparison, so the null is robust to moderate and not strong selection. Individual-level data on those patients were not covered by the ethics approval and were not extracted. The centre is single and the instrument would not hold where endoscopy runs 24 h a day. With 22 deaths, mortality analyses are underpowered for moderate effects and every causal-layer interval is wide. Consultation timestamps clustered narrowly, with 15.0% at exactly 2.00 h. Post-endoscopy stay is derived and rests on 388 of 413 episodes, although the slope analysis avoids the coupling problem. Transfusion could not be timed relative to endoscopy. Ascertainment relied on records. The pivotal trial timed endoscopy from consultation, not arrival [4]; our consultation-anchored analysis changed nothing.

## 5. Conclusions

In this single-centre cohort, the day of arrival, together with a weekday endoscopy schedule, largely determined the time to endoscopy in non-variceal upper gastrointestinal bleeding. Delay beyond 24 h was associated with no detectable difference in 30-day mortality, rebleeding, repeat endoscopy, intensive care admission or a composite of adverse events. This finding carries a qualification, however: 22 deaths leave a moderate effect in either direction possible, and patients who never reach endoscopy are not represented. The one consistent consequence of delay was a longer admission. Each day of waiting added one day of stay, with no measurable change in the time from endoscopy to discharge. The delay arose after gastroenterology consultation in the gap between the decision to scope and an available list, rather than in the emergency department. Shortening it is therefore a question of endoscopy capacity. The case for doing so rests on bed occupancy rather than on survival.

## Figures and Tables

**Table 1:** Baseline characteristics of the cohort and of the three endoscopy timing groups.

Characteristic	Urgent (<12 h), *n* = 96	Early (12–24 h), *n* = 129	Elective (>24 h), *n* = 188	*p* Value
Age, years	70.5 (60.8–80.0)	73.0 (62.0–80.0)	73.0 (63.0–81.0)	0.680
Systolic blood pressure, mmHg	113 (105–124)	117 (101–130)	114 (100–130)	0.964
Heart rate, beats/min	88 (77–100)	89 (80–103)	88 (78–104)	0.553
Shock index	0.77 (0.66–0.90)	0.78 (0.65–0.95)	0.78 (0.64–0.93)	0.887
Glasgow Coma Scale	15 (15–15)	15 (15–15)	15 (15–15)	0.056
Admission haemoglobin, g/dL	9.2 (7.5–10.9)	8.6 (7.0–10.9)	9.0 (7.1–11.1)	0.566
Blood urea, mg/dL	91.0 (63.8–129.8)	83.0 (57.0–132.0)	80.5 (51.0–127.2)	0.380
Creatinine, mg/dL	1.00 (0.80–1.38)	1.10 (0.80–1.57)	1.04 (0.80–1.53)	0.745
International normalised ratio	1.20 (1.09–1.31)	1.20 (1.08–1.34)	1.17 (1.05–1.34)	0.382
Albumin, g/dL	3.65 (3.10–4.00)	3.50 (3.10–4.00)	3.50 (3.00–3.90)	0.432
Platelets, ×10^3^/µL	216.5 (166.2–279.2)	237.0 (192.0–298.0)	228.0 (170.0–286.0)	0.225
Charlson comorbidity index	4.5 (3.0–5.0)	5.0 (3.0–6.0)	4.0 (3.0–6.0)	0.845
Number of comorbidities	1 (0–2)	1 (0–2)	1 (0–2)	0.929
Male sex	71 (74.0)	93 (72.1)	122 (64.9)	0.206
Haematemesis	33 (34.4)	40 (31.0)	50 (26.6)	0.373
Melena	65 (67.7)	91 (70.5)	139 (73.9)	0.527
Haematochezia	9 (9.4)	15 (11.6)	30 (16.0)	0.251
Syncope	19 (19.8)	27 (20.9)	37 (19.7)	0.960
Altered consciousness	14 (14.6)	10 (7.8)	13 (6.9)	0.086
ASA physical status ≥ III	55 (57.3)	73 (56.6)	120 (63.8)	0.356
Any antithrombotic agent	46 (47.9)	56 (43.4)	79 (42.0)	0.634
Proton pump inhibitor use	11 (11.5)	11 (8.5)	32 (17.0)	0.076
Cirrhosis	2 (2.1)	4 (3.1)	5 (2.7)	1.000
Active malignancy	5 (5.2)	7 (5.4)	14 (7.4)	0.677

Continuous variables are median (interquartile range) and categorical variables are *n* (%).

**Table 2:** The interval from emergency department arrival to endoscopy and its two components.

Interval or Event	Median (IQR) or *n* (%)	Episodes, *n*
Arrival to consultation request	2.00 (1.67–2.33)	413
Consultation request to endoscopy	19.88 (11.08–46.67)	413
Arrival to endoscopy	22.13 (13.23–49.47)	413
Endoscopy within 24 h	225 (54.5)	413
Endoscopy in weekday working hours	406 (98.3)	413

Values are median (interquartile range) in hours for the three intervals and *n* (%) for the two counts. The consultation-to-endoscopy interval exceeded the arrival-to-consultation interval on the paired Wilcoxon signed-rank test (*p* < 0.001). Weekday working hours are 08:00 to 17:00, Monday to Friday.

**Table 3:** Crude clinical outcomes by endoscopy timing group.

Outcome	All Episodes, *n* = 413	Urgent (<12 h), *n* = 96	Early (12–24 h), *n* = 129	Elective (>24 h), *n* = 188	*p* Value	Adjusted *p* Value
A. Binary outcomes, *n* (%) of the group						
30-day mortality	22 (5.3)	4 (4.2)	5 (3.9)	13 (6.9)	0.420	primary outcome, unadjusted
In-hospital mortality	19 (4.6)	3 (3.1)	4 (3.1)	12 (6.4)	0.351	0.390
Rebleeding	49 (11.9)	12 (12.5)	15 (11.6)	22 (11.7)	0.976	0.976
Repeat endoscopy	80 (19.4)	25 (26.0)	24 (18.6)	31 (16.5)	0.151	0.251
Red-cell transfusion	193 (46.7)	33 (34.4)	63 (48.8)	97 (51.6)	0.019	0.096
Intensive care admission	116 (28.1)	25 (26.0)	30 (23.3)	61 (32.4)	0.177	0.253
Surgery	1 (0.2)	0 (0.0)	0 (0.0)	1 (0.5)	1.000	not applicable
Interventional radiology	1 (0.2)	0 (0.0)	0 (0.0)	1 (0.5)	1.000	not applicable
Composite outcome	105 (25.4)	30 (31.2)	30 (23.3)	45 (23.9)	0.323	0.390
B. Continuous outcomes, median (IQR), all 413 episodes						
Red-cell units transfused	0 (0–2)	0 (0–2)	0 (0–2)	1 (0–2)	0.037	0.124
Total length of stay, days	4 (3–7)	3 (1–6)	4 (2–6)	5 (4–8)	<0.001	<0.001
Intensive care days	0 (0–2)	0 (0–1)	0 (0–0)	0 (0–2)	0.122	0.244
C. Stay decomposition, median (IQR), the 388 episodes with a computable post-endoscopy interval (80 urgent, 129 early, 179 elective)						
Total length of stay, days	5.00 (3.00–7.00)	4.00 (2.00–6.25)	4.00 (2.00–6.00)	5.00 (4.00–8.50)	<0.001	exploratory
Post-endoscopy length of stay, days	3.15 (1.43–5.67)	3.81 (1.88–6.03)	3.31 (1.49–5.35)	2.47 (1.25–5.25)	0.061	0.152

Panel A gives events with the percentage of the group in parentheses; the first data column gives all episodes. The seven outcomes in the confirmatory family (in-hospital mortality to composite outcome, plus red-cell units and total stay in panel B) carry Benjamini–Hochberg adjusted *p* values; 30-day mortality is the primary outcome and is unadjusted. Surgery and interventional radiology occurred once each and were not tested in the adjusted family. Panel C repeats total stay and reports post-endoscopy stay, derived as total stay minus the wait, on the same 388 episodes; 25 episodes with a recorded stay shorter than the wait (16 urgent, 0 early, and 9 elective; 16 with a recorded stay of 0 days) are excluded from that panel only. *p* values are chi-square or Fisher exact tests for binary outcomes and Kruskal–Wallis tests for continuous outcomes.

**Table 4:** Pairwise comparisons of every outcome between the three endoscopy timeframes in the whole cohort.

Outcome	Contrast	First Group, Events/*n* (%) or Median (IQR)	Second Group, Events/*n* (%) or Median (IQR)	Risk Difference, Percentage Points (95% CI)	Odds Ratio (95% CI) or Shift in Days (95% CI)	*p* Value	Holm-Adjusted *p* Value
30-day mortality	Urgent vs. early	4/96 (4.2)	5/129 (3.9)	0.3 (−4.9 to 5.5)	1.08 (0.21–5.16)	1.000	1.000
	Elective vs. early	13/188 (6.9)	5/129 (3.9)	3.0 (−1.9 to 8.0)	1.84 (0.60–6.76)	0.326	0.979
	Urgent vs. elective	4/96 (4.2)	13/188 (6.9)	−2.7 (−8.1 to 2.6)	0.59 (0.14–1.97)	0.436	0.979
In-hospital mortality	Urgent vs. early	3/96 (3.1)	4/129 (3.1)	0.0 (−4.6 to 4.6)	1.01 (0.14–6.11)	1.000	1.000
	Elective vs. early	12/188 (6.4)	4/129 (3.1)	3.3 (−1.3 to 7.9)	2.13 (0.63–9.26)	0.296	0.888
	Urgent vs. elective	3/96 (3.1)	12/188 (6.4)	−3.3 (−8.2 to 1.7)	0.47 (0.08–1.82)	0.400	0.888
Rebleeding	Urgent vs. early	12/96 (12.5)	15/129 (11.6)	0.9 (−7.8 to 9.5)	1.09 (0.44–2.63)	0.839	1.000
	Elective vs. early	22/188 (11.7)	15/129 (11.6)	0.1 (−7.1 to 7.3)	1.01 (0.48–2.18)	1.000	1.000
	Urgent vs. elective	12/96 (12.5)	22/188 (11.7)	0.8 (−7.3 to 8.9)	1.08 (0.46–2.40)	0.849	1.000
Repeat endoscopy	Urgent vs. early	25/96 (26.0)	24/129 (18.6)	7.4 (−3.6 to 18.5)	1.54 (0.77–3.06)	0.195	0.389
	Elective vs. early	31/188 (16.5)	24/129 (18.6)	−2.1 (−10.7 to 6.4)	0.86 (0.46–1.63)	0.652	0.652
	Urgent vs. elective	25/96 (26.0)	31/188 (16.5)	9.6 (−0.7 to 19.8)	1.78 (0.93–3.37)	0.060	0.181
Red-cell transfusion	Urgent vs. early	33/96 (34.4)	63/129 (48.8)	−14.5 (−27.3 to −1.6)	0.55 (0.31–0.98)	0.041	0.081
	Elective vs. early	97/188 (51.6)	63/129 (48.8)	2.8 (−8.4 to 14.0)	1.12 (0.70–1.79)	0.649	0.649
	Urgent vs. elective	33/96 (34.4)	97/188 (51.6)	−17.2 (−29.1 to −5.3)	0.49 (0.28–0.84)	0.008	0.024
Intensive care admission	Urgent vs. early	25/96 (26.0)	30/129 (23.3)	2.8 (−8.6 to 14.2)	1.16 (0.60–2.24)	0.641	0.641
	Elective vs. early	61/188 (32.4)	30/129 (23.3)	9.2 (−0.7 to 19.1)	1.58 (0.93–2.74)	0.079	0.236
	Urgent vs. elective	25/96 (26.0)	61/188 (32.4)	−6.4 (−17.4 to 4.6)	0.73 (0.40–1.31)	0.279	0.557
Composite outcome	Urgent vs. early	30/96 (31.2)	30/129 (23.3)	8.0 (−3.8 to 19.8)	1.50 (0.79–2.84)	0.223	0.606
	Elective vs. early	45/188 (23.9)	30/129 (23.3)	0.7 (−8.8 to 10.2)	1.04 (0.59–1.83)	1.000	1.000
	Urgent vs. elective	30/96 (31.2)	45/188 (23.9)	7.3 (−3.8 to 18.4)	1.44 (0.80–2.58)	0.202	0.606
Total length of stay, days	Urgent vs. early	3.00 (1.00–6.00), *n* = 96	4.00 (2.00–6.00), *n* = 129	not applicable	−1.00 (−2.00 to −0.00) days	0.027	0.027
	Elective vs. early	5.00 (4.00–8.00), *n* = 188	4.00 (2.00–6.00), *n* = 129	not applicable	1.00 (1.00 to 2.00) days	<0.001	<0.001
	Urgent vs. elective	3.00 (1.00–6.00), *n* = 96	5.00 (4.00–8.00), *n* = 188	not applicable	−2.00 (−3.00 to −1.00) days	<0.001	<0.001
Post-endoscopy length of stay, days	Urgent vs. early	3.81 (1.88–6.03), *n* = 80	3.31 (1.49–5.35), *n* = 129	not applicable	0.48 (−0.39 to 1.36) days	0.217	0.261
	Elective vs. early	2.47 (1.25–5.25), *n* = 179	3.31 (1.49–5.35), *n* = 129	not applicable	−0.37 (−1.03 to 0.14) days	0.131	0.261
	Urgent vs. elective	3.81 (1.88–6.03), *n* = 80	2.47 (1.25–5.25), *n* = 179	not applicable	0.79 (0.08 to 1.58) days	0.034	0.101

Each outcome is compared between every pair of timing groups within the full analysis set. For binary outcomes, the cells give events/episodes (%), the risk difference in percentage points with a Wald 95% CI, and the odds ratio with an exact 95% CI from Fisher’s test; *p* values are Fisher exact. For the two stay outcomes, the cells give the median (IQR) with the group size; the effect is the Hodges–Lehmann shift in days with its 95% CI, and *p* values are Wilcoxon rank-sum. Post-endoscopy stay uses the 388 episodes on which it is computable. Holm adjustment is applied within each outcome across its three contrasts; this table is descriptive and sits outside the prespecified confirmatory family of Table 3.

**Table 5:** Effect estimates for the principal outcomes under five analytic approaches, with each timing group compared with early endoscopy.

Outcome	Contrast	Crude Estimate (95% CI)	Multivariable-Adjusted Estimate (95% CI)	Inverse Probability of Treatment-Weighted Estimate (95% CI)	Propensity-Score-Matched Estimate (95% CI)
30-day all-cause mortality, odds ratio	Urgent (<12 h) versus early (12–24 h)	1.08 (0.28–4.13)	0.69 (0.13–3.71)	0.77 (0.19–3.08)	1.00 (0.19–5.29)
30-day all-cause mortality, odds ratio	Elective (>24 h) versus early (12–24 h)	1.84 (0.64–5.30)	1.42 (0.50–4.03)	1.23 (0.41–3.73)	2.92 (1.04–8.17)
Red-cell transfusion, odds ratio	Urgent (<12 h) versus early (12–24 h)	0.55 (0.32–0.95)	0.48 (0.26–0.88)	0.60 (0.34–1.04)	0.57 (0.31–1.03)
Red-cell transfusion, odds ratio	Elective (>24 h) versus early (12–24 h)	1.12 (0.71–1.75)	1.32 (0.76–2.30)	1.21 (0.77–1.92)	1.25 (0.73–2.14)
Composite outcome, odds ratio	Urgent (<12 h) versus early (12–24 h)	1.50 (0.83–2.72)	1.33 (0.71–2.48)	1.38 (0.75–2.53)	1.69 (0.82–3.49)
Composite outcome, odds ratio	Elective (>24 h) versus early (12–24 h)	1.04 (0.61–1.76)	0.96 (0.56–1.65)	0.96 (0.56–1.65)	1.05 (0.58–1.88)
Total length of stay, incidence rate ratio	Urgent (<12 h) versus early (12–24 h)	not modelled without adjustment	0.85 (0.64–1.13)	not applicable	not applicable
Total length of stay, incidence rate ratio	Elective (>24 h) versus early (12–24 h)	not modelled without adjustment	1.40 (1.14–1.73)	not applicable	not applicable
Post-endoscopy length of stay, incidence rate ratio	Urgent (<12 h) versus early (12–24 h)	not modelled without adjustment	1.14 (0.84–1.54)	not applicable	not applicable
Post-endoscopy length of stay, incidence rate ratio	Elective (>24 h) versus early (12–24 h)	not modelled without adjustment	1.05 (0.80–1.38)	not applicable	not applicable

Odds ratio models are adjusted for the ABC score; the composite additionally adjusted for a high-risk Forrest class and endoscopic therapy; and transfusion adjusted for admission haemoglobin, antithrombotic use and sex, with standard errors clustered on patient by generalised estimating equations. Length-of-stay rows are incidence rate ratios from negative binomial models adjusted for the ABC score with patient-clustered standard errors and are neither weighted nor matched; post-endoscopy rows use the 388 episodes with a computable interval. Propensity-score matching is one-to-one within each pair of groups (urgent versus early, 88 pairs; elective versus early, 124 pairs) with standard errors clustered on matched pairs; the elective-versus-early matched mortality estimate rests on 11 deaths against 4 and is discussed in the text and Table S14. Crude odds ratios are unadjusted logistic estimates with Wald confidence intervals.

**Table 6:** Validity and results of the natural experiment using arrival on a Friday or Saturday as an instrument for endoscopy timing.

Panel	Item	Friday or Saturday Arrival, *n* = 90	Other Days, *n* = 323	*p* Value
A. Exposure shift produced by the instrument	Urgent (<12 h), %	11.1	26.6	tested in the first stage, panel B
	Early (12–24 h), %	1.1	39.6	tested in the first stage, panel B
	Elective (>24 h), %	87.8	33.7	tested in the first stage, panel B
B. First stage and baseline severity by instrument (exclusion restriction)	First-stage coefficient, additional hours to endoscopy	29.85	reference category	<0.001
	First-stage F statistic, hours to endoscopy	39.02	not applicable	<0.001
	First-stage coefficient, absolute increase in elective (>24 h) proportion	0.54	reference category	<0.001
	First-stage F statistic, elective (>24 h) status	103.16	not applicable	<0.001
	Age, years (median)	75.00	72.00	0.704
	ABC score (median)	4.00	4.00	0.818
	Glasgow-Blatchford score (median)	10.50	11.00	0.603
	ASA physical status (median)	3.00	3.00	0.632
	Charlson comorbidity index (median)	5.00	4.00	0.881
	Admission Rockall score (median)	2.00	3.00	0.364
	MAP(ASH) score (median)	3.00	3.00	0.615
	Admission haemoglobin, g/dL (median)	9.00	8.90	0.527
	AIMS65 score (median)	1.00	1.00	0.547
C. Reduced form (instrument to outcome)	30-day all-cause mortality, %	3.3	5.9	0.435
	In-hospital mortality, %	3.3	5.0	0.776
	Rebleeding, %	13.3	11.5	0.762
	Repeat endoscopy, %	20.0	19.2	0.984
	Red-cell transfusion, %	47.8	46.4	0.916
	Intensive care unit admission, %	25.6	28.8	0.637
	Surgery, %	0.0	0.3	1.000
	Interventional radiology, %	0.0	0.3	1.000
	Composite outcome, %	26.7	25.1	0.866
	Total length of stay, days (median), all 413 episodes	5.00	4.00	0.054
	Post-endoscopy length of stay, days (median), 388 episodes with a computable interval	2.25, *n* = 88	3.32, *n* = 300	0.003
	Red-cell units transfused (median)	0.00	0.00	0.702
D. Two-stage least squares (elective >24 h versus earlier endoscopy)	Total length of stay, days	1.048 (−3.123 to 5.220)	not applicable	0.623
	30-day all-cause mortality, risk difference	−0.047 (−0.132 to 0.037)	not applicable	0.275
	Red-cell transfusion, risk difference	0.025 (−0.192 to 0.241)	not applicable	0.823

The instrument is arrival on a Friday or Saturday. In panel D, the third column carries the two-stage least squares estimate with its 95% confidence interval and the instrument column headings do not apply. Percentages in panels A and C are within the instrument group. The instrument group sizes in the column headings apply to every row except the post-endoscopy row of panel C, which is confined to the 388 episodes with a computable post-endoscopy interval and carries its own group size in each cell.

## Data Availability

The data for this study were obtained from the same patient cohort as the residency (medical specialty) thesis of Lokman Bayram, completed at the Department of Emergency Medicine, Bolu Abant İzzet Baysal University Faculty of Medicine. Patient identifiers were removed before any analysis, and the dataset was held within the institution throughout. No text, table, or figure is duplicated from either source. The data presented in this study are available on request from the corresponding author. The data are not publicly available because they consist of retrospectively collected clinical records held within the institution; the ethics approval (protocol code 2026/249, 9 June 2026) covers analysis of the de-identified dataset within the institution only, and any release to third parties requires separate institutional approval. The analysis code that produced every reported quantity is available from the corresponding author on request.

## Supplementary Materials

The following supporting information can be downloaded at https://www.mdpi.com/article/10.3390/jcm15207820/s1. Table S1. Pre-endoscopy risk scores by endoscopy timing group; Table S2a. Exploratory subgroup analyses of endoscopy timing and outcome; Table S2b. Tests for the interaction between endoscopy timing and the subgroup variables; Table S3. Prespecified sensitivity analyses with p values for the four principal outcomes under each definition of the exposure or of the analysis set; Table S4. Outcomes under the four-tier timing scheme of the 2026 European guideline; Table S5. Timing of endoscopy by day of emergency department arrival; Table S6. Covariate balance before and after inverse probability of treatment weighting; Table S7. Firth penalised logistic models for the rare outcomes; Table S8. Endoscopic diagnosis in the analysis set; Table S9. Forrest classification of the index lesion; Table S10. Type of endoscopic therapy applied; Table S11. Agreement between the two operational definitions of the ABC and AIMS65 scores and their discrimination for 30-day mortality; Table S12. Covariate balance in the propensity-matched sample; Table S13. Effect estimates for every confirmatory outcome with and without patient-level clustering by generalised estimating equations; Table S14. Propensity-matched and inverse probability weighted estimates for each pair of timing groups, urgent versus early and elective versus early; Table S15. Covariate balance in the elective-versus-early propensity-matched sample; Table S16. Mathematical coupling of the post-endoscopy interval to the wait and alternative specifications of the stay outcome that do not rely on the derived interval; Table S17. Tipping-point and scenario analysis for the 369 patients who did not undergo endoscopy.

## Abbreviations

2SLS	two-stage least squares
ABC	age, blood tests and comorbidities score
AIMS65	albumin, INR, mental status, systolic blood pressure, age > 65 score
ASA	American Society of Anesthesiologists
CI	confidence interval
ED	emergency department
ESGE	European Society of Gastrointestinal Endoscopy
GBS	Glasgow-Blatchford score
GEE	generalised estimating equations
HR	hazard ratio
ICU	intensive care unit
INR	international normalised ratio
IPTW	inverse probability of treatment weighting
IQR	interquartile range
IRR	incidence rate ratio
MAP(ASH)	mental status, ASA, pulse, albumin, systolic blood pressure, haemoglobin score
NVUGIB	non-variceal upper gastrointestinal bleeding
OR	odds ratio
SMD	standardised mean difference
STROBE	Strengthening the Reporting of Observational Studies in Epidemiology

## References

[1] Gralnek I.M., Morris J., Laursen S.B., Camus M., Tziatzios G., Debels L.K., Nigam G.B., Erőss B., Goetz M., Forbes N. (2026). Endoscopic diagnosis and management of peptic ulcer bleeding: European Society of Gastrointestinal Endoscopy (ESGE) Guideline—Update 2026. Endoscopy.

[2] Gralnek I.M., Stanley A.J., Morris A.J., Camus M., Lau J., Lanas A., Laursen S.B., Radaelli F., Papanikolaou I.S., Cúrdia Gonçalves T. (2021). Endoscopic diagnosis and management of nonvariceal upper gastrointestinal hemorrhage (NVUGIH): European Society of Gastrointestinal Endoscopy (ESGE) Guideline—Update 2021. Endoscopy.

[3] Laine L., Barkun A.N., Saltzman J.R., Martel M., Leontiadis G.I. (2021). ACG Clinical Guideline: Upper Gastrointestinal and Ulcer Bleeding. Am. J. Gastroenterol..

[4] Lau J.Y.W., Yu Y., Tang R.S.Y., Chan H.C.H., Yip H.C., Chan S.M., Luk S.W.Y., Wong S.H., Lau L.H.S., Lui R.N. (2020). Timing of Endoscopy for Acute Upper Gastrointestinal Bleeding. N. Engl. J. Med..

[5] Merola E., Michielan A., de Pretis G. (2021). Optimal timing of endoscopy for acute upper gastrointestinal bleeding: A systematic review and meta-analysis. Intern. Emerg. Med..

[6] Guo C.L.T., Wong S.H., Lau L.H.S., Lui R.N.S., Mak J.W.Y., Tang R.S.Y., Yip T.C.F., Wu W.K.K., Wong G.L.H., Chan F.K.L. (2022). Timing of endoscopy for acute upper gastrointestinal bleeding: A territory-wide cohort study. Gut.

[7] Bai L., Jiang W., Cheng R., Dang Y., Min L., Zhang S. (2023). Does Early Endoscopy Affect the Clinical Outcomes of Patients with Acute Nonvariceal Upper Gastrointestinal Bleeding? A Systematic Review and Meta-Analysis. Gut Liver.

[8] El-Dallal M., Walradt T.J., Stein D.J., Khrucharoen U., Feuerstein J.D. (2022). Pros and Cons of Performing Early Endoscopy in Geriatric Patients Admitted with Non-variceal Upper Gastrointestinal Bleeding: Analysis of the US National Inpatient Database. Dig. Dis. Sci..

[9] Aziz M., Dasari C.S., Zafar Y., Fatima R., Haghbin H., Alyousif Z.A., Lee-Smith W., Perisetti A., Nawras A., Garg S. (2021). Does timing of endoscopy affect outcomes in patients with upper gastrointestinal bleeding: A systematic review and meta-analysis. Eur. J. Gastroenterol. Hepatol..

[10] Stasik K., Ferenc K., Partyka K., Kilisiński W., Filip R. (2025). Impact of Urgent Versus Early Endoscopy on Outcomes in Acute Upper Gastrointestinal Bleeding: A Retrospective Study. Can. J. Gastroenterol. Hepatol..

[11] Marmo C., Bucci C., Soncini M., Riccioni M.E., Marmo R., on behalf of the Gised Study Group (2025). Defining and Evaluating the Impact of Bleeding Severity on Time to Endoscopy and Mortality Risk: A Prospective Multicenter Cohort Study. J. Clin. Med..

[12] Austin P.C., Stuart E.A. (2015). Moving towards best practice when using inverse probability of treatment weighting (IPTW) using the propensity score to estimate causal treatment effects in observational studies. Stat. Med..

[13] Hernán M.A., Robins J.M. (2006). Instruments for causal inference: An epidemiologist’s dream?. Epidemiology.

[14] Baiocchi M., Cheng J., Small D.S. (2014). Instrumental variable methods for causal inference. Stat. Med..

[15] Hernán M.A., Robins J.M. (2016). Using Big Data to Emulate a Target Trial When a Randomized Trial Is Not Available. Am. J. Epidemiol..

[16] Siau K., Hodson J., Ingram R., Baxter A., Widlak M.M., Sharratt C., Baker G.M., Troth T., Hicken B., Tahir F. (2019). Time to endoscopy for acute upper gastrointestinal bleeding: Results from a prospective multicentre trainee-led audit. United Eur. Gastroenterol. J..

[17] Güven İ.E., Başpınar B., Durak M.B., Yüksel İ. (2023). Comparison of urgent and early endoscopy for acute non-variceal upper gastrointestinal bleeding in high-risk patients. Gastroenterol. Hepatol..

[18] von Elm E., Altman D.G., Egger M., Pocock S.J., Gøtzsche P.C., Vandenbroucke J.P. (2007). The Strengthening the Reporting of Observational Studies in Epidemiology (STROBE) statement: Guidelines for reporting observational studies. Lancet.

[19] Laursen S.B., Oakland K., Laine L., Bieber V., Marmo R., Redondo-Cerezo E., Dalton H.R., Ngu J., Schultz M., Soncini M. (2021). ABC score: A new risk score that accurately predicts mortality in acute upper and lower gastrointestinal bleeding: An international multicentre study. Gut.

[20] Jimenez-Rosales R., Lopez-Tobaruela J.M., Lopez-Vico M., Ortega-Suazo E.J., Martinez-Cara J.G., Redondo-Cerezo E. (2023). Performance of the New ABC and MAP(ASH) Scores in the Prediction of Relevant Outcomes in Upper Gastrointestinal Bleeding. J. Clin. Med..

[21] Yavuz A., Karabulut Ü., Ebik B., Akkuzu M.Z., Bingöl F. (2026). Prognostic Value of the Neutrophil Percentage-to-Albumin Ratio in Acute Non-Variceal Upper Gastrointestinal Bleeding. J. Clin. Med..

[22] Cagir Y., Durak M.B., Yuksel I. (2024). Optimal endoscopy timing in elderly patients presenting with acute non-variceal upper gastrointestinal bleeding. BMC Gastroenterol..

[23] Foroughian M., Ravaghi A., Habibzadeh S.R., Goshayeshi L., Abbasi Shaye Z., Ziyaei M., Talebi Doluee M. (2025). Impact of Timing of Endoscopy on Mortality in Non-variceal Upper Gastrointestinal Bleeding: A Retrospective Cohort Study. Galen. Med. J..

[24] Gorur M., Satar S., Gulen M., Acehan S., Sevdimbas S., Nigiz K. (2026). Timing of endoscopy and its impact on prognosis in patients admitted to the emergency department with upper gastrointestinal bleeding. Ir. J. Med. Sci..

[25] Eid E., Elliott A., Rockey D.C. (2026). Time to endoscopy and its effect on outcomes in acute nonvariceal and variceal upper gastrointestinal bleeding. J. Investig. Med..

[26] Tejedor-Tejada J., Hermida B., Camblor C., Emara M., Youssef Mohamed S., Perez-Pariente J.M. (2025). Timing of endoscopy in high-risk patients with acute upper gastrointestinal bleeding: Results of a multicenter international cohort study. Gastroenterol. Hepatol..

[27] Laursen S.B., Leontiadis G.I., Stanley A.J., Møller M.H., Hansen J.M., Schaffalitzky de Muckadell O.B. (2017). Relationship between timing of endoscopy and mortality in patients with peptic ulcer bleeding: A nationwide cohort study. Gastrointest. Endosc..

[28] Kumar N.L., Cohen A.J., Nayor J., Claggett B.L., Saltzman J.R. (2017). Timing of upper endoscopy influences outcomes in patients with acute nonvariceal upper GI bleeding. Gastrointest. Endosc..

[29] Bezati S., Ventoulis I., Verras C., Boultadakis A., Bistola V., Sbyrakis N., Fraidakis O., Papadamou G., Fyntanidou B., Parissis J. (2025). Major Bleeding in the Emergency Department: A Practical Guide for Optimal Management. J. Clin. Med..

[30] Yaghnam I., Kochhar S., Razjouyan H. (2026). Impact of Scheduled Outpatient Endoscopy Procedures on Inpatient Endoscopy Procedures: Prospective Analysis from a Single Tertiary Care Center. Dig. Dis. Sci..

[31] Jacobs C.C., Jaber J.F., Ladna M., Ponniah S., Madhok I., Ruiz N., Podeschi H., Gesiotto F., Khan W., Moon N. (2021). Factors Associated With Inpatient Endoscopy Delay and its Impact on Hospital Length-of-Stay and 30-Day Readmission. Clin. Gastroenterol. Hepatol..

[32] Nedjat-Shokouhi B., Glynn M., Denton E.R.E., Greenfield S.M. (2017). Provision of out-of-hours services for acute upper gastrointestinal bleeding in England: Results of the 2014-2015 BSG/NHS England national survey. Frontline Gastroenterol..

[33] Gupta A., Agarwal R., Ananthakrishnan A.N. (2018). “Weekend Effect” in Patients With Upper Gastrointestinal Hemorrhage: A Systematic Review and Meta-analysis. Am. J. Gastroenterol..

[34] Sanders D.S., Perry M.J., Jones S.G., McFarlane E., Johnson A.G., Gleeson D.C., Lobo A.J. (2004). Effectiveness of an upper-gastrointestinal haemorrhage unit: A prospective analysis of 900 consecutive cases using the Rockall score as a method of risk standardisation. Eur. J. Gastroenterol. Hepatol..

[35] Parisio L., Cuccia G., Privitera G., Castaldo F., Carbone L., Minordi L.M., Pugliese D. (2026). Treat-to-Target in Ulcerative Colitis: How Soon Is Now?. J. Clin. Med..

[36] Pavel C., Diculescu M.M., Stepan A.E., Constantinescu G., Sandru V., Ţieranu C.G., Tomescu L., Constantinescu A., Patoni C., Plotogea O.M. (2024). Considering Histologic Remission in Ulcerative Colitis as a Long-Term Target. J. Clin. Med..

[37] Guan J.L., Wang G., Fang D., Han Y.Y., Wang M.R., Tian D.A., Li P.Y. (2022). Does off-hours endoscopic hemostasis affect outcomes of nonvariceal upper gastrointestinal bleeding?. J. Comp. Eff. Res..

[38] Martino A., Di Serafino M., Orsini L., Giurazza F., Fiorentino R., Crolla E., Campione S., Molino C., Romano L., Lombardi G. (2023). Rare causes of acute non-variceal upper gastrointestinal bleeding: A comprehensive review. World J. Gastroenterol..

[39] Jo S.Y., Noh J.H., Cha B., Ahn J.Y., Oh S.P., Seo J.Y., Na H.K., Lee J.H., Jung K.W., Kim D.H. (2023). Clinical outcomes of Dieulafoy’s lesion compared with peptic ulcer in upper gastrointestinal bleeding. J. Gastroenterol. Hepatol..

[40] Mulita F., Kouroupi M., Verras G.I., Mitropoulou A.M., Bolanaki H., Tzimagiorgis I., Giatromanolaki A., Karayiannakis A.J. (2026). Life-Threatening Gastrointestinal Bleeding Revealing a Rare Coexistence of Ampullary Ganglioneuroma and Pancreatic Neuroendocrine Tumor: A Case Report. Diagnostics.

[41] Sohail A., Vankayala U.S.A., George B., Javor J., Deeb L. (2026). Absence of a Weekend Effect on In-Hospital Mortality in Upper Gastrointestinal Bleeding: A Multisite Study from an Integrated Health System. Dig. Dis. Sci..

[42] Soncini M., Occhipinti V., Zullo A., Marmo R. (2022). No evidence of “weekend effect” in upper gastrointestinal bleeding in Italy: Data from a nationwide prospective registry. Eur. J. Gastroenterol. Hepatol..

[43] Qiu J., Xia Y., Ding R., Ouyang Q., Wang L., Huang Y., Chen S., Yang Z., Shu X., Pan X. (2026). Experience of the endoscopist mediates the association between weekend effect with rebleeding after endoscopic treatment for Dieulafoy’s lesion. Surg. Endosc..

