# Supplementary material for: Day of Arrival as a Determinant of Endoscopy Timing in Non-Variceal Upper Gastrointestinal Bleeding: A Natural-Experiment Cohort Study of Clinical Outcomes and Length of Stay
Source: J Clin Med. 2026 Oct 10;15(20):7820. doi: 10.3390/jcm15207820 (PMC13705092; doi:10.3390/jcm15207820)
Supplement: Supplementary file 1 [file jcm-15-07820-s001.zip › jcm-4586612-supplementary.pdf]

Journal of Clinical Medicine

MDPI

# Supplementary Materials

**Day of Arrival as a Determinant of Endoscopy Timing in Non-Variceal Upper Gastrointestinal Bleeding: A Natural-Experiment Cohort Study of Clinical Outcomes and Length of Stay**

Table S1. Pre-endoscopy risk scores by endoscopy timing group.

| Score | Urgent (<12 h), *n* = 96 | Early (12-24 h), *n* = 129 | Elective (>24 h), *n* = 188 | p value |
|---|---|---|---|---|
| Glasgow–Blatchford score | 11 (8–12.25) | 11 (8–13) | 11 (7–13) | 0.395 |
| AIMS65 score | 1 (0–2) | 1 (0–2) | 1 (1–2) | 0.392 |
| ABC score | 4 (2–6.25) | 4 (3–6) | 4 (3–7) | 0.556 |
| MAP(ASH) score | 2 (1–3) | 3 (2–3) | 3 (1–3) | 0.211 |
| T-Score | 10 (8–11) | 10 (8–10) | 9 (8–11) | 0.468 |
| Admission Rockall score | 2 (1–4) | 3 (1–4) | 3 (1–4) | 0.230 |
| Charlson comorbidity index | 4.5 (3–5) | 5 (3–6) | 4 (3–6) | 0.845 |
| Values are median (interquartile range). | | | | |

Table S2a. Exploratory subgroup analyses of endoscopy timing and outcome.

| Subgroup | Episodes in subgroup, *n* | Outcome | Urgent (<12 h), % | Early (12–24 h), % | Elective (>24 h), % | p value |
|---|---|---|---|---|---|---|
| Age <65 years | 121 | 30-day all-cause mortality, % | 0.0 | 0.0 | 3.7 | 0.506 |
| Age ≥ 65 years | 292 | 30-day all-cause mortality, % | 6.2 | 5.4 | 8.2 | 0.763 |
| Age <65 years | 121 | Red-cell transfusion, % | 25.8 | 27.8 | 44.4 | 0.128 |
| Age ≥ 65 years | 292 | Red-cell transfusion, % | 38.5 | 57.0 | 54.5 | 0.049 |
| Glasgow–Blatchford score <12 | 243 | Composite outcome, % | 33.3 | 21.6 | 17.4 | 0.059 |
| Glasgow–Blatchford score ≥ 12 | 170 | Composite outcome, % | 27.8 | 25.5 | 32.9 | 0.629 |
| ABC score <8 | 338 | 30-day all-cause mortality, % | 3.7 | 0.9 | 2.7 | 0.385 |
| ABC score ≥ 8 | 75 | 30-day all-cause mortality, % | 7.1 | 21.1 | 21.4 | 0.568 |
| Low-risk Forrest class | 318 | Rebleeding, % | 10.6 | 5.9 | 10.6 | 0.406 |

| | | | | | | |
|---|---|---|---|---|---|---|
| High-risk Forrest class | 95 | Rebleeding, % | 16.7 | 32.1 | 16.2 | 0.230 |

Across the whole cohort the timing groups comprise 96 urgent, 129 early and 188 elective episodes. Subgroup sizes are shown for each stratum.

Table S2b. Tests for interaction between endoscopy timing and the subgroup variables.

| Outcome | Effect modifier | p value for interaction |
|---|---|---|
| 30-day all-cause mortality | Age ≥ 65 years versus <65 years | 0.421 |
| Red-cell transfusion | Age ≥ 65 years versus <65 years | 0.208 |
| Composite outcome | Glasgow–Blatchford score ≥ 12 versus <12 | 0.278 |
| 30-day all-cause mortality | ABC score ≥ 8 versus <8 | 0.048 |
| Rebleeding | High-risk versus low-risk Forrest class | 0.066 |

Interaction terms are between timing group and the effect modifier. No p value is corrected for multiplicity.

Table S3. Prespecified sensitivity analyses. p values for the four principal outcomes under each definition of the exposure or of the analysis set.

| Analysis | Episodes, *n* | p value, red-cell transfusion | p value, 30-day all-cause mortality | p value, total length of stay | p value, post-endoscopy length of stay |
|---|---|---|---|---|---|
| Main analysis, full cohort | 413 | 0.019 | 0.420 | <0.001 | 0.061 |
| First attack only | 387 | 0.041 | 0.640 | <0.001 | 0.080 |
| Daytime arrivals only | 406 | 0.022 | 0.407 | <0.001 | 0.046 |
| ESGE 2026 four-tier | 413 | 0.046 | 0.638 | <0.001 | 0.098 |
| Guo 2022 scheme | 413 | 0.172 | 0.502 | <0.001 | 0.145 |
| Consultation as time zero | 413 | 0.054 | 0.561 | <0.001 | 0.054 |
| Arrivals in 2020 and 2021 excluded | 344 | 0.050 | 0.596 | <0.001 | 0.045 |

Each row repeats the primary between-group comparison under the stated definition or restriction. The episode count is the size of the sensitivity set; within each set the post-endoscopy column is tested on the episodes of that set for which a post-endoscopy interval is computable, so it matches the primary post-endoscopy analysis in Table 3, where that subset is 388 of the 413 episodes. The 2020 and 2021 restriction removes 69 episodes from the two years in which hospital activity in Türkiye was most affected by the COVID-19 pandemic; in that subset the adjusted odds ratio for 30-day mortality, elective versus early, was 1.47 (0.46-4.65) and the incidence rate ratio for total stay was 1.31 (1.09-1.58).

Table S4. Outcomes under the four-tier timing scheme of the 2026 European guideline.

| Outcome | ≤ 6 h, *n* = 56 | 6–12 h, *n* = 40 | 12–24 h, *n* = 129 | >24 h, *n* = 188 | p value |
|---|---|---|---|---|---|
| 30-day all-cause mortality, % | 5.4 | 2.5 | 3.9 | 6.9 | 0.549 |
| Red-cell transfusion, % | 35.7 | 32.5 | 48.8 | 51.6 | 0.046 |

| | | | | | |
|---|---|---|---|---|---|
| Composite outcome, % | 28.6 | 35.0 | 23.3 | 23.9 | 0.429 |
| Total length of stay, days (median), all 413 episodes | 3.0 | 4.0 | 4.0 | 5.0 | <0.001 |
| Post-endoscopy length of stay, days (median), 388 episodes with a computable interval | 3.8, *n* = 44 | 4.1, *n* = 36 | 3.3, *n* = 129 | 2.5, *n* = 179 | 0.098 |

Tiers follow the four-category European Society of Gastrointestinal Endoscopy timing scheme. The tier sizes in the column headings sum to 413 episodes and apply to every row except the post-endoscopy row, which is confined to the 388 episodes with a computable post-endoscopy interval and carries its own tier size in each cell.

Table S5. Timing of endoscopy by day of emergency department arrival.

| Day of emergency department arrival | Episodes, *n* | Hours to endoscopy, median | Endoscopy within 24 h, % | Assigned to elective (>24 h) group, % |
|---|---|---|---|---|
| Monday | 80 | 17.8 | 68 | 32.5 |
| Tuesday | 59 | 18.1 | 71 | 28.8 |
| Wednesday | 54 | 17.6 | 69 | 31.5 |
| Thursday | 71 | 16.3 | 75 | 25.4 |
| Friday | 50 | 64.8 | 22 | 78.0 |
| Saturday | 40 | 48.1 | 0 | 100.0 |
| Sunday | 59 | 24.3 | 47 | 52.5 |

Episode counts sum to 413. Endoscopy is performed on weekdays at this centre.

Table S6. Covariate balance before and after inverse probability of treatment weighting.

| Covariate | Covariate type | Maximum absolute difference between timing groups before weighting | Maximum absolute difference between timing groups after weighting |
|---|---|---|---|
| Age, years | Continuous | 0.098 | 0.036 |
| Male sex | Binary | 0.091 | 0.016 |
| ABC score | Continuous | 0.131 | 0.034 |
| Glasgow–Blatchford score | Continuous | 0.164 | 0.005 |
| ASA physical status ≥ III | Binary | 0.072 | 0.007 |
| Charlson comorbidity index | Continuous | 0.082 | 0.035 |
| Systolic blood pressure, mmHg | Continuous | 0.045 | 0.018 |
| Admission hemoglobin, g/dL | Continuous | 0.150 | 0.015 |
| Hematemesis | Binary | 0.078 | 0.005 |
| Any antithrombotic agent | Binary | 0.059 | 0.017 |

Covariates are listed in the order entered into the propensity model. Continuous covariates are compared as standardized mean differences and binary covariates as absolute differences in proportions. Each value is the largest of the three pairwise comparisons between timing groups.

Table S7. Firth penalised logistic models for the rare outcomes.

| Outcome | Comparison | Odds ratio (95% CI) | p value |
|---|---|---|---|
| 30-day all-cause mortality | Urgent (<12 h) versus early (12–24 h) | 0.73 (0.16–2.93) | 0.658 |
| | Elective (>24 h) versus early (12–24 h) | 1.36 (0.48–4.28) | 0.573 |
| | ABC score, per one point | 1.46 (1.27–1.69) | <0.001 |
| In-hospital mortality | Urgent (<12 h) versus early (12–24 h) | 0.65 (0.12–3.06) | 0.586 |
| | Elective (>24 h) versus early (12–24 h) | 1.53 (0.51–5.40) | 0.461 |
| | ABC score, per one point | 1.47 (1.27–1.73) | <0.001 |

Estimates are from Firth penalized logistic regression, which corrects the small-event bias of the maximum likelihood models. Blank cells in the first column continue the outcome named in the row above.

Table S8. Endoscopic diagnosis in the analysis set.

| Endoscopic diagnosis | Episodes, *n* | Percentage of 413 episodes |
|---|---|---|
| Peptic ulcer | 167 | 40.4 |
| Erosive gastritis or duodenitis | 130 | 31.5 |
| No bleeding source identified | 38 | 9.2 |
| Malignancy | 27 | 6.5 |
| Other | 21 | 5.1 |
| Esophagitis | 15 | 3.6 |
| Dieulafoy lesion | 7 | 1.7 |
| Cameron lesion | 3 | 0.7 |
| Angiodysplasia | 2 | 0.5 |
| Mallory-Weiss tear | 2 | 0.5 |
| Esophageal ulcer | 1 | 0.2 |
| One diagnosis per episode. Counts sum to 413 episodes. | | |

Table S9. Forrest classification of the index lesion.

| Forrest class | Episodes, *n* | Percentage of 413 episodes |
|---|---|---|
| Forrest III | 64 | 15.5 |
| Forrest IIa | 37 | 9.0 |
| Forrest IIb | 6 | 1.5 |
| Forrest IIc | 9 | 2.2 |
| Forrest Ia | 6 | 1.5 |
| Forrest Ib | 46 | 11.1 |
| Not applicable | 245 | 59.3 |
| High-risk classes combined (Ia, Ib, IIa, IIb) | 95 | 23.0 |

Not applicable denotes an endoscopic finding for which the Forrest classification does not apply. Counts of the individual classes sum to 413 episodes; the combined high-risk row is a subtotal of those classes.

https://doi.org/10.3390/jcm15207820

Table S10. Type of endoscopic therapy applied.

| Endoscopic therapy | Episodes, *n* | Percentage of 413 episodes |
| --- | --- | --- |
| Other | 2 | 0.5 |
| Clip placement | 61 | 14.8 |
| Combined injection and clip placement | 33 | 8.0 |
| Injection sclerotherapy | 19 | 4.6 |
| None | 298 | 72.2 |
| Any endoscopic therapy applied | 115 | 27.8 |
| Successful haemostasis among episodes treated endoscopically | 108 | 93.9 (percentage of 115 treated episodes) |

The individual therapy rows sum to 413 episodes. The last two rows are subtotals: episodes receiving any therapy, and successful haemostasis within those episodes.

Table S11. Agreement between the two operational definitions of the ABC and AIMS65 scores and their discrimination for 30-day mortality.

| Score definition compared | Episodes classified identically, *n* | Episodes assessed, *n* | Area under the receiver operating characteristic curve, workbook definition | Area under the receiver operating characteristic curve, published definition |
| --- | --- | --- | --- | --- |
| ABC score, workbook creatinine thresholds versus published thresholds | 349 | 413 | 0.855 | 0.852 |
| AIMS65 score, systolic blood pressure <90 mmHg versus ≤ 90 mmHg | 396 | 413 | 0.850 | 0.847 |

Both definitions were applied to the same 413 episodes and the two areas under the curve were compared for the same outcome as in the primary score analysis.

Table S12. Covariate balance in the propensity-matched sample.

| Covariate | Mean, matched urgent (<12 h) episodes | Mean, matched early (12–24 h) episodes | Standardized mean difference | Variance ratio | Mean difference in empirical cumulative distribution | Maximum difference in empirical cumulative distribution | Standardized pair distance |
| --- | --- | --- | --- | --- | --- | --- | --- |
| Propensity score | 0.429 | 0.427 | 0.032 | 1.027 | 0.012 | 0.068 | 0.047 |
| Age, years | 69.557 | 68.898 | 0.039 | 1.177 | 0.030 | 0.114 | 0.977 |
| Male sex | 0.739 | 0.705 | 0.078 | not applicable, binary covariate | 0.034 | 0.034 | 0.958 |
| ABC score | 4.670 | 4.602 | 0.021 | 1.140 | 0.019 | 0.034 | 1.005 |
| Glasgow–Blatchford score | 10.670 | 10.523 | 0.038 | 0.868 | 0.020 | 0.080 | 1.004 |

| | | | | | | | |
|---|---|---|---|---|---|---|---|
| ASA physical status ≥ III | 0.580 | 0.591 | -0.023 | not applicable, binary covariate | 0.011 | 0.011 | 1.080 |
| Charlson comorbidity index | 4.386 | 4.318 | 0.034 | 0.766 | 0.027 | 0.057 | 1.056 |
| Systolic blood pressure, mmHg | 116.193 | 114.841 | 0.073 | 0.667 | 0.038 | 0.091 | 1.236 |
| Admission hemoglobin, g/dL | 8.983 | 8.972 | 0.004 | 0.952 | 0.031 | 0.091 | 1.037 |
| Hematemesis | 0.318 | 0.352 | -0.072 | not applicable, binary covariate | 0.034 | 0.034 | 0.790 |
| Any antithrombotic agent | 0.466 | 0.409 | 0.114 | not applicable, binary covariate | 0.057 | 0.057 | 0.978 |

Matching produced 88 pairs (176 episodes). Covariates are listed in the order entered into the propensity model. A variance ratio is not defined for binary covariates.

Table S13. Effect estimates for every confirmatory outcome with and without patient-level clustering by generalised estimating equations.

| **Outcome** | **Events, *n*** | **Contrast** | **Covariates** | **Odds ratio, ordinary logistic** | **95% CI** | **p value** | **Odds ratio, GEE** | **95% CI, cluster-robust** | **p value, GEE** |
|---|---|---|---|---|---|---|---|---|---|
| 30-day mortality | 22 | Urgent vs early | ABC score | 0.69 | 0.16–3.05 | 0.629 | 0.69 | 0.13–3.71 | 0.670 |
| | 22 | Elective vs early | ABC score | 1.42 | 0.47–4.33 | 0.538 | 1.42 | 0.50–4.03 | 0.510 |
| In-hospital mortality | 19 | Urgent vs early | ABC score | 0.60 | 0.11–3.22 | 0.554 | 0.60 | 0.09–4.06 | 0.603 |
| | 19 | Elective vs early | ABC score | 1.63 | 0.49–5.50 | 0.427 | 1.63 | 0.53–5.03 | 0.392 |
| Rebleeding | 49 | Urgent vs early | ABC score, high-risk Forrest class, endoscopic therapy, antithrombotic use | 0.95 | 0.41–2.18 | 0.901 | 0.95 | 0.42–2.13 | 0.898 |
| | 49 | Elective vs early | ABC score, high-risk Forrest class, | 0.99 | 0.48–2.03 | 0.981 | 0.99 | 0.49–1.99 | 0.981 |

| | | | | | | | | | |
|---|---|---|---|---|---|---|---|---|---|
| | | | endoscopic therapy, antithrombotic use | | | | | | |
| Repeat endoscopy | 80 | Urgent vs early | ABC score, high-risk Forrest class, endoscopic therapy | 1.36 | 0.71–2.63 | 0.355 | 1.36 | 0.71–2.61 | 0.349 |
| | 80 | Elective vs early | ABC score, high-risk Forrest class, endoscopic therapy | 0.84 | 0.46–1.54 | 0.574 | 0.84 | 0.46–1.53 | 0.571 |
| Red-cell transfusion | 193 | Urgent vs early | ABC score, admission hemoglobin, antithrombotic use, male sex | 0.48 | 0.24–0.94 | 0.033 | 0.48 | 0.26–0.88 | 0.019 |
| | 193 | Elective vs early | ABC score, admission hemoglobin, antithrombotic use, male sex | 1.32 | 0.75–2.31 | 0.336 | 1.32 | 0.76–2.30 | 0.330 |
| Intensive care admission | 116 | Urgent vs early | ABC score | 1.11 | 0.58–2.10 | 0.756 | 1.11 | 0.57–2.13 | 0.762 |
| | 116 | Elective vs early | ABC score | 1.48 | 0.87–2.51 | 0.150 | 1.48 | 0.85–2.57 | 0.169 |
| Composite outcome | 105 | Urgent vs early | ABC score, high-risk Forrest class, endoscopic therapy | 1.33 | 0.71–2.48 | 0.373 | 1.33 | 0.71–2.48 | 0.373 |

https://doi.org/10.3390/jcm15207820

| | | | | | | | | | |
|---|---|---|---|---|---|---|---|---|---|
| | 105 | Elective vs early | ABC score, high-risk Forrest class, endoscopic therapy | 0.96 | 0.55–1.68 | 0.895 | 0.96 | 0.56–1.65 | 0.892 |
| Total length of stay | 413 | Urgent vs early | ABC score (negative binomial, incidence rate ratio) | 0.849 | | 0.135 | 0.849 | 0.636–1.133 | 0.266 |
| | | Elective vs early | ABC score (negative binomial, incidence rate ratio) | 1.402 | | <0.001 | 1.402 | 1.140–1.725 | 0.001 |
| Total length of stay, 388-episode subset | 388 | Urgent vs early | ABC score (negative binomial, incidence rate ratio) | 1.013 | | 0.907 | 1.013 | 0.767–1.336 | 0.929 |
| | | Elective vs early | ABC score (negative binomial, incidence rate ratio) | 1.451 | | <0.001 | 1.451 | 1.177–1.789 | <0.001 |
| Post-endoscopy length of stay | 388 | Urgent vs early | ABC score (negative binomial, incidence rate ratio) | 1.139 | | 0.338 | 1.139 | 0.842–1.541 | 0.397 |
| | | Elective vs early | ABC score (negative binomial, incidence | 1.054 | | 0.639 | 1.054 | 0.802–1.384 | 0.707 |

https://doi.org/10.3390/jcm15207820

| | | | |
|---|---|---|---|
| | | rate ratio) | |

Generalised estimating equations with an independence working correlation and patient as the cluster, so that the 23 patients contributing 26 repeat episodes do not count as independent observations. Point estimates equal the ordinary logistic estimates; only the standard errors change. The three stay rows are negative binomial models with cluster-robust (patient) standard errors, on the 413 episodes for total stay and on the 388 episodes with a computable interval for the post-endoscopy rows; the ordinary CI column is left blank for those rows because the point estimate is shared.

Table S14. Propensity-matched and inverse probability weighted estimates for each pair of timing groups, urgent versus early and elective versus early.

| **Comparison** | **Outcome** | **Matched episodes, *n*** | **Events in matched sample, *n*** | **Matched estimate** | **95% CI, matched** | **p value, matched** | **Weighted estimate** | **95% CI, weighted** | **p value, weighted** |
|---|---|---|---|---|---|---|---|---|---|
| Urgent vs early | 30-day mortality | 176 | 6 | 1.00 | 0.19–5.29 | 1.000 | 1.00 | 0.25–3.90 | 0.996 |
| | Red-cell transfusion | 176 | 72 | 0.57 | 0.31–1.03 | 0.064 | 0.58 | 0.33–1.00 | 0.051 |
| | Rebleeding | 176 | 21 | 1.11 | 0.44–2.82 | 0.820 | 1.07 | 0.47–2.43 | 0.870 |
| | Composite outcome | 176 | 47 | 1.69 | 0.82–3.49 | 0.152 | 1.49 | 0.82–2.72 | 0.193 |
| | Intensive care admission | 176 | 44 | 1.13 | 0.63–2.03 | 0.685 | 1.22 | 0.65–2.26 | 0.537 |
| | Total length of stay | 176 | NA | 0.92 | 0.68–1.25 | 0.609 | 0.89 | 0.67–1.18 | 0.431 |
| | Post-endoscopy length of stay | 163 | NA | 1.21 | 0.89–1.65 | 0.227 | 1.19 | 0.89–1.60 | 0.229 |
| Elective vs early | 30-day mortality | 248 | 15 | 2.92 | 1.04–8.17 | 0.041 | 1.17 | 0.39–3.54 | 0.783 |
| | Red-cell transfusion | 248 | 127 | 1.25 | 0.73–2.14 | 0.408 | 1.20 | 0.76–1.90 | 0.434 |
| | Rebleeding | 248 | 32 | 1.15 | 0.53–2.51 | 0.716 | 0.95 | 0.46–1.96 | 0.889 |
| | Composite outcome | 248 | 59 | 1.05 | 0.58–1.88 | 0.882 | 0.94 | 0.54–1.61 | 0.816 |
| | Intensive care | 248 | 69 | 1.56 | 0.86–2.84 | 0.147 | 1.45 | 0.86–2.46 | 0.161 |

| | | | | | | | | |
|---|---|---|---|---|---|---|---|---|
| admission | | | | | | | | |
| Total length of stay | 248 | NA | 1.43 | 1.13–1.81 | 0.003 | 1.46 | 1.16–1.82 | 0.001 |
| Post-endoscopy length of stay | 241 | NA | 1.09 | 0.79–1.49 | 0.610 | 1.12 | 0.83–1.51 | 0.472 |

One-to-one nearest-neighbour propensity matching (caliper 0.2 standard deviations of the logit) and inverse probability of treatment weighting, each pair of timing groups analysed separately with the same ten covariates as the main propensity model (age, sex, ABC and Glasgow-Blatchford scores, ASA class III or higher, Charlson index, systolic blood pressure, admission hemoglobin, hematemesis, antithrombotic use). Urgent versus early: 88 pairs, largest absolute standardised mean difference after matching 0.114 and after weighting 0.013. Elective versus early: 124 pairs (all 129 early episodes were eligible controls, 5 remained unmatched), largest standardised difference 0.091 matched and 0.011 weighted. Binary outcomes are odds ratios with standard errors clustered on matched pair (matched) or robust to weighting (weighted); the two stay rows are incidence rate ratios. The matched elective-versus-early estimate for 30-day mortality rests on 11 deaths against 4 (9 discordant pairs against 2; exact McNemar p = 0.065; conditional logistic odds ratio 4.50, 95% CI 0.97 to 20.83; Firth penalised odds ratio adjusted for the ABC score 3.09, 95% CI 0.91 to 12.64) and is not reproduced by the weighted estimate on the same covariates.

Table S15. Covariate balance in the elective-versus-early propensity-matched sample.

| Covariate | Mean, matched elective (>24 h) episodes | Mean, matched early (12–24 h) episodes | Standardized mean difference |
|---|---|---|---|
| Age | 68.516 | 69.621 | −0.073 |
| Male sex | 0.694 | 0.71 | −0.034 |
| ABC score | 4.556 | 4.54 | 0.005 |
| Glasgow-Blatchford score | 10.5 | 10.863 | −0.091 |
| ASA class III or higher | 0.597 | 0.573 | 0.05 |
| Charlson index | 4.266 | 4.347 | −0.037 |
| Systolic blood pressure | 117.444 | 116.653 | 0.032 |
| Admission hemoglobin | 8.818 | 8.838 | −0.007 |
| Hematemesis | 0.282 | 0.306 | −0.055 |

Covariate balance in the 124 matched elective-versus-early pairs. Binary covariates are proportions. All absolute standardized differences are below 0.10.

Table S16. Mathematical coupling of the post-endoscopy interval to the wait, and alternative specifications of the stay outcome that do not rely on the derived interval.

| Item | Estimate (95% CI) or value | Note |
|---|---|---|
| A. Correlation structure on the 388 episodes with a computable post-endoscopy interval | | |
| Spearman rho, wait versus total stay | 0.306 | |
| Spearman rho, wait versus post-endoscopy stay | −0.131 | |
| Pearson r, wait versus total stay | 0.240 | |
| Pearson r, wait versus post-endoscopy stay | 0.008 | |

| | | |
|---|---|---|
| Pearson r expected from coupling alone, if total stay were independent of the wait | −0.226 | |
| Variance of the wait, days squared | 3.127 | |
| Variance of total stay, days squared | 58.224 | |
| Covariance of wait and total stay, days squared | 3.236 | |
| B. Slope of measured total stay on measured wait, days of stay per day of waiting (uses no derived variable) | | |
| Ordinary least squares, ABC-adjusted, cluster-robust (*n* = 413) | 1.029 (0.596 to 1.462) | p versus slope of 1: 0.896 |
| Ordinary least squares, unadjusted (*n* = 413) | 1.093 (0.669 to 1.517) | p versus slope of 1: 0.667 |
| Median regression, ABC-adjusted (*n* = 413) | 0.884 (0.665 to 1.103) | p versus slope of 1: 0.299 |
| Ordinary least squares, waits of 5 days or less, ABC-adjusted (*n* = 395) | 0.745 (0.263 to 1.228) | p versus slope of 1: 0.3 |
| Two-stage least squares, Fri/Sat instrument, ABC-adjusted (*n* = 413) | 0.476 (−1.289 to 2.242) | p versus slope of 1: 0.561 |
| C. Alternative specifications of the stay outcome | | |
| Total stay adjusted for ABC and continuous wait (hours), all 413, Urgent vs early | 0.895 (0.724–1.107) | IRR; p = 0.308 |
| Total stay adjusted for ABC and continuous wait (hours), all 413, Elective vs early | 1.092 (0.888–1.342) | IRR; p = 0.405 |
| Total stay: wait itself (per 24 h) in the same model, Wait, per 24 h | 1.115 (1.058–1.175) | IRR; p <0.001 |
| Post-endoscopy stay floored at 0, all 413 retained, Urgent vs early | 0.955 (0.726–1.256) | IRR; p = 0.741 |
| Post-endoscopy stay floored at 0, all 413 retained, Elective vs early | 1.000 (0.793–1.261) | IRR; p = 0.998 |
| Discharge hazard after endoscopy, Cox, cluster by patient, 388, Urgent vs early | 0.878 (0.680–1.134) | HR (>1 = faster discharge); p = 0.319 |
| Discharge hazard after endoscopy, Cox, cluster by patient, 388, Elective vs early | 1.029 (0.819–1.293) | HR (>1 = faster discharge); p = 0.808 |
| Two-stage least squares, elective versus other timing, Friday or Saturday instrument, total length of stay (*n* = 413) | 1.05 (−3.12 to 5.22) days | p = 0.623 |
| Two-stage least squares, elective versus other timing, Friday or Saturday instrument, post-endoscopy length of stay (*n* = 388) | −1.79 (−6.10 to 2.53) days | p = 0.417 |
| D. The 25 episodes without a computable post-endoscopy interval | | |
| Urgent, *n* | 16 | |
| Early, *n* | 0 | |
| Elective, *n* | 9 | |

| | |
|---|---|
| Recorded total stay of 0 days, *n* | 16 |
| Median Glasgow-Blatchford score, excluded episodes | 8 |
| Median Glasgow-Blatchford score, retained 388 episodes | 11 |
| Median ABC score, excluded episodes | 4 |
| Median ABC score, retained 388 episodes | 4 |
| Deaths within 30 days, excluded episodes | 0 |
| Endoscopic therapy applied, excluded episodes, *n* | 4 |

Panel A: post-endoscopy stay is total stay minus the wait, so if total stay were unrelated to the wait, the derived variable would correlate negatively with the wait by construction (the coupling value in the fifth row). The observed correlation is close to zero, which is what a one-to-one carry-over of the wait into total stay produces. Panel B tests that carry-over directly on two measured quantities: a slope of 1 means each day of waiting adds one day of admission and no more; a slope above 1 would indicate that waiting also prolongs recovery, a slope below 1 that part of the wait is absorbed. Panel C: the first model conditions total stay on the continuous wait; the second keeps all 413 episodes by setting negative derived intervals to zero; the third models the time from endoscopy to discharge as a hazard, clustered on patient, where a hazard ratio above 1 means faster discharge. Panel D characterises the excluded episodes: they are predominantly urgent same-day discharges with lower Glasgow-Blatchford scores and no deaths, so their exclusion removes short stays from the urgent group and works against, not for, the finding that post-endoscopy stay is not longer after delay.

Table S17. Tipping-point and scenario analysis for the 369 patients who did not undergo endoscopy.

| Item | Value | Column 3 | Column 4 | Column 5 |
|---|---|---|---|---|
| A. Observed data and tipping points | | | | |
| Observed 30-day deaths, urgent / early / elective | 4/5/13 | | | |
| Group sizes, urgent / early / elective | 96/129/188 | | | |
| Additional deaths that would have to enter the elective group for the three-group comparison to reach p < 0.05 | 8 | | | |
| The same number as a percentage of the 369 patients not scoped | 2.2 | | | |
| Additional elective deaths for the elective-versus-early 95% CI to exclude 1 | 8 | | | |
| Additional deaths among Friday or | 9 | | | |

| | | | | |
|---|---|---|---|---|
| Saturday arrivals for the reduced-form mortality difference to reach p < 0.05 | | | | |
| Observed 30-day mortality, Friday or Saturday versus other arrival days | 3.3% vs 5.9% | | | |
| B. Scenarios for the 369 patients not scoped, assuming 80 arrived on Friday or Saturday and 289 on other days (the 21.8% share observed in the scoped cohort) | | | | |
| 30-day mortality assumed among the 369 | Relative risk of death, Friday or Saturday versus other-day arrival, among the 369 | Combined mortality, Friday or Saturday arrivals | Combined mortality, other days | p value for the combined reduced-form comparison |
| 2% | 1 | 2.9% | 4.1% | 0.653 |
| 5% | 1 | 4.1% | 5.4% | 0.694 |
| 10% | 1 | 6.5% | 7.8% | 0.625 |
| 15% | 1 | 8.8% | 10.1% | 0.665 |
| 2% | 1.5 | 2.9% | 3.9% | 0.652 |
| 5% | 1.5 | 4.7% | 5.2% | 1 |
| 10% | 1.5 | 8.2% | 7.4% | 0.743 |
| 15% | 1.5 | 11.2% | 9.5% | 0.56 |
| 2% | 2 | 3.5% | 3.9% | 1 |
| 5% | 2 | 5.9% | 5.1% | 0.697 |
| 10% | 2 | 9.4% | 7% | 0.324 |
| 15% | 2 | 13.5% | 9% | 0.084 |
| 2% | 3 | 3.5% | 3.8% | 1 |
| 5% | 3 | 6.5% | 4.7% | 0.43 |
| 10% | 3 | 11.8% | 6.4% | 0.031 |
| 15% | 3 | 16.5% | 8% | 0.002 |

Panel A asks how many deaths, unobserved because the patients never reached endoscopy, would have to be added to the delayed group or to the Friday or Saturday arrivals for the reported null comparisons to become significant. Panel B combines the 413 scoped episodes with the 369 patients who were not scoped under assumed values for their mortality and for the excess mortality of weekend arrivals among them; each cell is the reduced-form comparison of 30-day mortality by arrival day that would then be observed in all 782 patients. The comparison remains non-significant unless weekend arrivals who were not scoped died at least twice as often as weekday arrivals who were not scoped and overall mortality among the unscoped exceeded 10%, that is, unless weekend arrival was itself lethal in the patients who did not reach endoscopy. Individual-level data on the 369 were not extracted (see Methods 2.2).
